# Non-Curcuminoids from Turmeric and Their Potential in Cancer Therapy and Anticancer Drug Delivery Formulations

**DOI:** 10.3390/biom9010013

**Published:** 2019-01-02

**Authors:** Akhila Nair, Augustine Amalraj, Joby Jacob, Ajaikumar B. Kunnumakkara, Sreeraj Gopi

**Affiliations:** 1 R&D Centre, Aurea Biolabs (P) Ltd., Kerala 682311, India; rd@plantlipids.com (A.N.); amalraj.a@plantlipids.com (A.A.); joby.jacob@plantlipids.com (J.J.); 2 Department of Biosciences and Bioengineering, Indian Institute of Technology, Guwahati 781 039, India; kunnumakkara@iitg.ernet.in

**Keywords:** turmeric, curcuminoids, non-curcuminoids, bioactive molecules, anticancer activity

## Abstract

Over the past decades curcuminoids have been extensively studied for their biological activities such as antiulcer, antifibrotic, antiviral, antibacterial, antiprotozoal, antimutagenic, antifertility, antidiabetic, anticoagulant, antivenom, antioxidant, antihypotensive, antihypocholesteremic, and anticancer activities. With the perception of limited toxicity and cost, these compounds forms an integral part of cancer research and is well established as a potential anticancer agent. However, only few studies have focused on the other bioactive molecules of turmeric, known as non-curcuminoids, which are also equally potent as curcuminoids. This review aims to explore the comprehensive potency including the identification, physicochemical properties, and anticancer mechanism inclusive of molecular docking studies of non-curcuminoids such as turmerones, elemene, furanodiene (FN), bisacurone, germacrone, calebin A (CA), curdione, and cyclocurcumin. An insight into the clinical studies of these curcumin-free compounds are also discussed which provides ample evidence that favors the therapeutic potential of these compounds. Like curcuminoids, limited solubility and bioavailability are the most fragile domain, which circumscribe further applications of these compounds. Thus, this review credits the encapsulation of non-curcuminoid components in diverse drug delivery systems such as co-crystals, solid lipid nanoparticles, liposomes, microspheres, polar-non-polar sandwich (PNS) technology, which help abolish their shortcomings and flaunt their ostentatious benefits as anticancer activities.

## 1. Introduction

Cancer is a fatal disease, which accounts for high mortality and low survival rate worldwide. The International Agency for Research on Cancer (IARC) has estimated that, by 2030, about 21.7 million recently-developed cancer cases and about 13 million cancer-related deaths would result because of the heterogeneous nature of cancerous cells and failure of most of the chemotherapeutic agents in achieving the desired objective [1,2]. Therefore, the universally perturbed issue of obtaining economical therapeutic efficiency and reduced side effects are creating a fervent interest among researchers on traditional medicines such as black pepper, garlic, ginger, turmeric etc. [3,4]. Continuous studies on the epidemiological patterns of these naturally occurring plant-derived medicines especially turmeric elucidate their imperative role in reducing the mortality by cancer [5,6,7].

Turmeric (*Curcuma longa* L.), a nutritional dietary spice from the Zingiberaceae family, has been extensively used for various applications. This rhizomatous herbaceous perennial plant found abundantly in Asia is renowned for its cosmetic, culinary, color, and medicinal applications for centuries [7,8]. Basically, its volatile oil and nonvolatile oleoresin constitutes of bioactive components, which are classified as diphenylheptanoids (nonvolatile) diphenylpentanoids (nonvolatile), phenyl propene (cinnamic acid type) derivatives (nonvolatile), turmeric oil containing terpenoids (volatile) [9]. Major diphenylheptanoids are curcumin, demethoxycurcumin, and bisdemethoxycurcumin, which are collectively called curcuminoids [10,11]. Non-curcuminoids can be defined as all the biologically active compounds other than curcuminoids, precisely, curcumin-free bioactive components of turmeric [12]. Curcuminoids have a mixed bag of biological activities such as anti-inflammatory, antiulcer, antiviral, antibacterial, antiprotozoal, antivenom and antioxidant. In addition, anticoagulant, antihypotensive, antihypocholesteremic, antifibrotic, antimutagenic, antifertility, and anticancer activities, which are well documented. Especially, the anticancer activities of curcuminoids are well established and evidenced with extensive studies on vivid disparate pathways to trigger apoptosis and cell cycle arrest are well established [13,14,15]. Recently, non-curcuminoids such as turmerones, elemene, bisacurone, curdione, cyclocurcumin, germacrone, furanodiene, curcumol, and calebin A were extensively studied, for their pharmacological activities such as anti-inflammatory, antioxidant, and anticancer activities. Researchers have specifically explained that non-curcuminoids have a broad spectrum of anticancer activities on different cell lines including intricate and drug resistant malignancies. Although these compounds promise to exhibit similar potency as curcuminoids, their anticancer studies are elusive and they still have to become a cynosure [12,16].

In this review, we comprehensively attempt to establish the basic attributes including the identification, composition, physicochemical properties, and various anticancer activities, including the mechanism of action and molecular docking studies of non-curcuminoids having equipotent synergistic effects as curcuminoids. Its therapeutic efficacy is also evinced by evaluating the successful clinical trials. Furthermore, we reviewed distinctive approaches that tend to ameliorate the solubility and bioavailability to aggrandize the anticancer effects of non-curcuminoids.

## 2. Identification of Non-Curcuminoids

Although turmeric was well recognized in Ayurveda and Chinese medicine from past decades, its constituents such as demethoxycurcumin, bismethoxycurcumin, elemene, bisacurone, calebin A, turmerone, germacrone, and so on, were identified later [12]. A breakthrough came in when it was officially first isolated from the rhizomes of *C. longa* by Vogel et al. and they named this compound ‘curcumin’ [17]. Another advancement made by Anderson et al. were the development of a novel technique using organic solvent for isolating curcumin from dried rhizomes of turmeric and ascertained presence of three components with thin layer chromatography [18]. Further, Milobedzka and Lampe et al. inveterate the feruloylmethyl structure of this compound [19] and, later, Lampe et al. revealed the synthesis of curcumin [20]. By the mid-twentieth century, the various potential biological activities of these polyphenolic compounds came into the limelight [21]. Kuttan et al. discovered the anticancer activities of these species in in vitro and in vivo models [22]. With the perception that there were other compounds in turmeric, Malingre et al. stated that turmeric contains p-cymene, β-sequiphellandrene, turmerone, and ar-turmerone [23]. Ohshiro et al. discovered and identified germacrone-13-al, pocurcumadiol, 4-hydroxybisabola-2, 10-diene-9-one, and 4-methoxy-S-hydroxybisabola-2, 10-diene-9-one [24]. Sesquiphellandrene, the active curcumin-free component of *C. longa* was also extensively studied [25]. Wang et al. isolated quinoline alkaloid and bisabolanes from the root of *C. longa* namely 2-(2-methyl-1-propenyl)-4, 6-dimethyl-7-hydroxyquinoline, 2, 5-dihydroxybisabola-3, 10-diene, 4, 5-dihydroxybisabola-2, 10-diene, bisacurone A, bisacurone B, bisacurone C, zingerone, dehydrozingerone, and turmeronol A [26]. Further, studies reported two more new sesquiterpenes from the ethanol extract of rhizome of *C. longa*, namely, 2-methoxy-5-hydroxybisabola-3, 10-diene-9-one, 2, 8 epoxy-5-hydroxybisabola-3, 10-diene-9-one, and one monoterpene: 2-(2, 5-dihydroxy-4-methylcyclohex-3-enyl) propanoic acid were also isolated for the first time from the genus *Curcuma* [27]. Even some lipid soluble fraction of this herb was isolated by solvent extraction method using non-polar organic solvents. These compounds were identified as turmerones and curlone, with a composition range of 50–60%, by gas chromatography-mass spectroscopy (GC-MS) and standardized by high-performance liquid chromatography (HPLC). These compounds were found light sensitive, but stable, at different temperatures and pH [28]. Kojima et al. discovered that the hydro distilled oil of turmeric contains α-turmerone, β-tumerone, and ar-turmerone by analysis through GC and GC-MS [29]. Besides, these non-curcuminoids were also found to be bioactive, exhibiting various anti-inflammatory, antioxidant, and anticancer activities. Itokawa et al., in 1985, established the anticancer activities of ar-turmerone by conducting a bioassay on sarcoma160 cells in mice [30]. The potential use of β-elemene as an anticancer drug was officially reported for the first time in China in 1993 [31].

## 3. Chemical Constituents of Non-Curcuminoids from Turmeric

Turmeric has a diverse chemical composition, principally containing approximately 235 terpenoids and phenolic compounds [12], which are obtained through various extraction procedures. To be specific, there are about 6–8 monomeric phenylpropenes, 22 diarylheptanoids and diarylpentanoids, 109 sesquiterpenes, 68 monoterpenes, four steroids, two alkaloids, five diterpenes, three triterpenoids, fatty acids, and 12 other compounds, along with sugar, proteins, carbohydrates, fiber, minerals, and resin that were isolated and identified [12,32].

One important class among them are diarylhepatanoids that include curcuminoids, are polyphenolic compounds containing curcumin (diferuloyl methane) and its two derivatives demethoxycurcumin, bisdemethoxycurcumin. Their distinct compositions were found to be in the ratio, namely curcumin (70–80%), demethoxycurcumin (18–20%) and bisdemethoxycurcumin (2–10%) [12], these diarylhepatanoids also include some curcumin-free compounds, such as tetrahydrocurcumin and cyclocurcumin. Other non-curcuminoids involve 109 sesquiterpenes [33], which are primarily found in turmeric oil, such as three elemanes, namely α-elemene, β-elemene, and γ-elemene; 54 bisabolanes, mainly α-turmerone, β-turmerone, ar-turmerone, bisacurone, and curlone; six germacranes, mainly germacrone, dehydrocurdione, β-germacene, and germacrone-13-al; along with seven guaianes, four selinanes, two caryophyllanes, aristolene, carbane, cedrane, bergamotane, and himachalane. From these sesquiterpenes, bisabolanes were found in the greatest abundance. In addition, few phenylpropenes, such as calebin A and ferulic acid, and some monoterpenoids, such as carvacrol and limonene, were also reported [12].

Hence, the enumerate curcumin-free compounds exhibiting distinctiveness in their composition, as well as biological activities, comprises turmerone, turmeronol, turmerine, himachalane, bergamotane, aristolene, silinanes, carbrane, cedrane, dihydrozingerone, sesquisabinane, sesquiphellandrene, curcumene, elemene, bisacurone, curdione, cyclocurcumin, germacrone, furanodiene, curcumol, calebin A, acrone, santalane, guaiane, curlone, carvacrol, limonene, tetrahydrocurcumin, hexahydrocurcumin, octahydrocurcumin, and ferulic acid [12].

## 4. Non-Curcuminoids as Anticancer Agents

**Figure 1:**
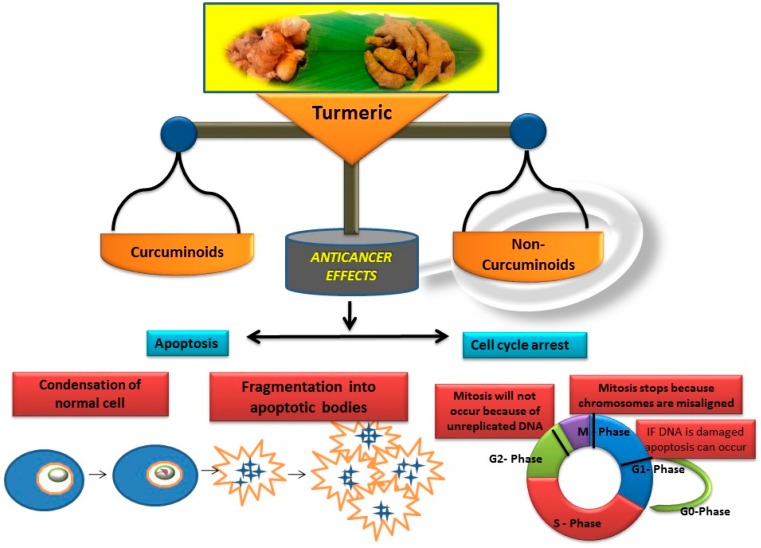
Schematic representation of anticancer activities of non-curcuminoids. Abbreviations: DNA, deoxyribonucleic acid.

Earlier researchers dynamically believed that curcuminoids are inimitable components of turmeric for anticancer activities. Although, many studies revealed that the other bioactive molecules, such as elemene, turmerones, curdione, carvacrol, germacrone, furanodiene, calebin A, bisacurone, curlone, allantone, ferulic acid, and dyhydrogingerone, have potential anticancer activities (Figure 1), a worldwide acknowledgement of these compounds are yet to be established so that they could be equally considered while formulating drug designs to cure cancer.

**Figure 2:**
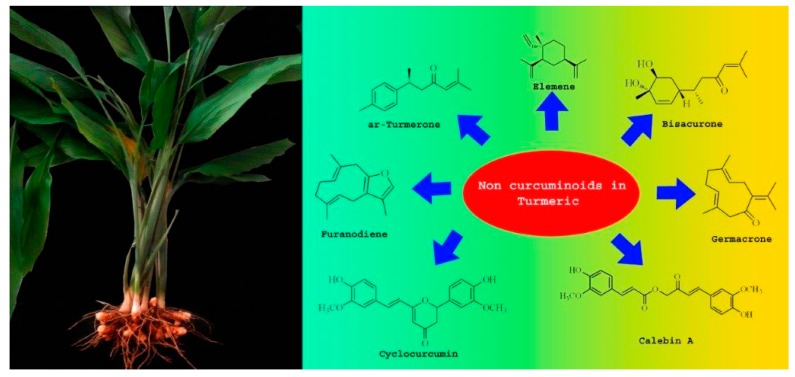
Chemical structures of selected non-curcuminoids from turmeric.

**Figure 3:**
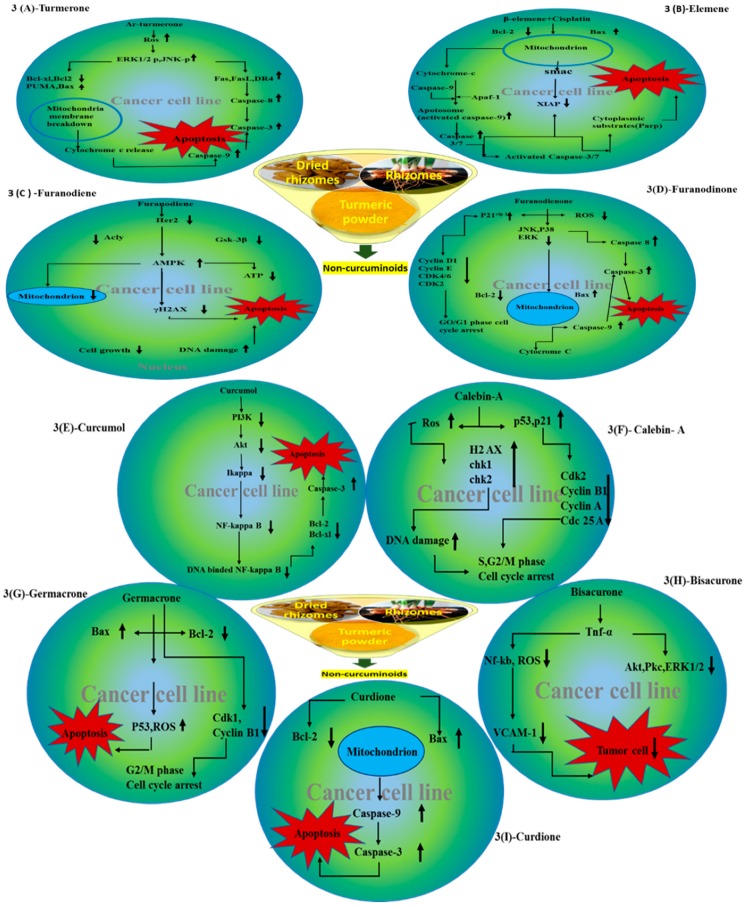
Mechanism of action of various non-curcuminoids anticancer pathways: (**A**) Ar-turmerone; (**B**) β-elemene; (**C**) furanodiene; (**D**) furanodinone; (**E**) curcumol; (**F**) calebin A; (**G**) germacrone; (**H**) bisacurone; and (**I**) curdione. Abbreviations: ACLY, ATP citrate lyase; Akt, protein kinase B, AMPK, adenosine monophosphate–activated protein kinase; Apaf, apoptotic protease activating factor; Ar–turmerone, aromatic turmerone; ATP, adenosine triphosphate; Bax, Bcl 2 associated X protein; Bcl, B cell lymphoma 2; cdc, cell division cycle; CDKI, cyclin–dependent kinases inhibitors; CHK2, checkpoint kinase 2; COX–2, cyclooxygenase –2; DNA, deoxyribonucleic acid; DR4, death receptor4; ERK, extracellular regulated kinase; FAS, first apoptosis signal; GSK, 3–glycogen synthase kinase; γH2AX, phosphorylation of H2A histone family member X on serine 139; Her 2, human epithelial growth factor receptor 2; JNK, c Jun N terminal kinase; NF–κB, nuclear factor kappa light chain enhancer of activated B cells; Parp, poly (ADP–ribose) polymerase; PKC, protein kinase C; PI3K, phosphoinositide 3-kinase; PUMA, p 53 up regulated modulator of apoptosis; ROS, reactive oxygen species; Smac, second mitochondria-derived activator of caspases; TNF, tumor necrosis factor; TPA, 12–O–tetradecanoylphorbol–13–acetate; VCAM, vascular cell adhesion molecules; Xiap, X-linked inhibitor of apoptosis protein.

The important non-curcuminoids and their physicochemical properties are given in Table 1. Researchers have accumulated data on the biological activities of non-curcuminoids and revealed that these components of turmeric have similar dynamic potential as curcuminoids and exhibits non-hepatotoxic, non-mutagenic, non-carcinogenic efficiency with the fewest side effects [16]. This review pioneers to collaborate all the foregoing extensive anticancer studies on some major non-curcuminoids (Figure 2) such as ar–turmerone, β–elemene, δ-elemene, furanodiene, furanodienone, curcumol, cyclocurcumin, calebin A, germacrone, bisacurone and curdione [33,34,35,36,37,38,39,40,41,42,43,44,45,46,47,48,49,50,51,52,53,54,55,56,57,58,59,60,61,62,63,64,65,66,67,68,69,70,71,72,73,74,75,76,77,78,79,80,81,82,83,84,85,86,87,88,89,90,91,92,93,94,95,96,97,98,99,100,101,102,103,104,105,106,107,108,109,110] (Table 2). Depending on different cancer cell lines there are various anticancer pathways enrolled in different non-curcuminoids and they are schematically represented in Figure 3.

### 4.1. Turmerones

Turmerones are one of the major sesquiterpenes derived from turmeric. They are further classified as α-turmerone, ar-turmerone, and β-turmerone, and among them ar-turmerone has shown to possess more potency as an anticancer agent. Nevertheless, Yue et al. explained the immunomodulatory and chemopreventive activities of α-turmerone and revealed its potency to be equivalent to curcuminoids. This study compared their potential in inhibiting of cell proliferation between the curcuminoids and α-turmerones on breast adenocarcinoma cell lines (MDA-MB-231). For this, propidium iodide staining, DNA fragmentation, and annexin-V were used to detect cell cycle phase, containing apoptotic cells, and its accumulation amount. It was observed that apoptosis was initiated due to the distortion caused to the mitochondria in the intrinsic pathway with activation in procaspase-3 cleavage in α-turmerone, whereas no effect was visualized when compared to curcuminoids. Further, this study explained that turmerones restrain the proliferation of cancer cells in a dose-dependent manner with half maximal inhibitory concentration (IC_50_) values ranging from 11.0 to 41.81 g/mL [33]. From this study it is clear that among turmerones, α-turmerone possess some anticancer activities. However, this component of non-curcuminoid has not yet gained enough significance. In addition to this, various anticancer activities of ar-turmerone registered until now are discussed further.

Ar-turmerone, (6S-2-methyl-6-(4-methylphenyl) hept-2-en-4-one), a sesquiterpene derived from *C. longa*, has been identified as a promising anticancer agent with distinct mechanism of action for different cancer cell lines. In this series, Lee et al. reported that ar-turmerone could bring DNA fragmentation (apoptosis) in human chronic myelogenous leukemia (K562), rat baselophilic leukemia (RBL-2H3), human histiocytic lymphoma (U937), and mouse lymphocytic leukemia (L1210) in a dose- and time-dependent manner [34]. The bioavailability of curcumin could be significantly increased by (α, ar) turmerones as explained by Yue et al. in human intestinal epithelial colorectal adenocarcinoma Caco-2 cells. It was reported that α-turmerone significantly reduced the p-glycoprotein (p-gp) with the upregulated level of multi-resistance protein gene 1 (MDR1), multi-resistance protein gene 2 (MDR2), and breast cancer resistance protein (BCRP). This was observed by the levels of messenger ribonucleic acid (mRNA) expression, rhodamine-123 accumulation, and efflux transport studies. These studies proved that α-turmerone and ar-turmerone have promising potential as anticancer drugs in multi-resistant cancer cells and colorectal cancer, respectively [35]. Further, ar-turmerone was reported to efficiently inhibited 12-O-tetradecanoylphorbol-13-acetate (TPA) induced invasion, colony formation, migration, stimulated matrix metalloproteinase (MMP-9), and cyclooxygenase-2 (COX-2) in breast cancer cells at non-cytotoxic concentrations. In addition, suppression noticed in MMP-9 and COX-2 promoter activation was due to the inhibition of its binding to nuclear factor kappa light chain enhancer of activated B-cells (NF–κB) in vivo via modulation of phosphoinositide 3-kinase/ protein kinase B (PI3K/Akt) and extracellular regulated kinase (ERK)1/2 signaling in breast cancer cells [36]. Other researchers, Yong-gang et al., examined the effects of purified ar-turmerone on murine dendritic cells (DC). This study utilized enzyme linked immunosorbent assay (ELISA), fluorescence-activated cell sorting (FACS), cyto-chemistry assay, and fluorescein isothiocyanate-dextran (FITC-dextran) bioassay for evaluating the effects of ar-turmerone on breast cancer cells. These tests showed that ar-turmerone increased the expression level of cluster of differentiation (CD40, CD80, CD83, and CD86), followed by the induction of phenotypic and functional maturation of DCs. Further, the treatment with ar-turmerone downregulated the acidic phosphatase (ACP) activity inside DC, promoting interleukin (IL-12) and tumor necrosis factor-α (TNF-α) production. They also reported that both α and β-turmerones exhibited antiproliferative activities in breast cells [37].

Moreover, the anticancer activities of ar-turmerone, isolated from the roots of *C. longa* and purified by supercritical carbon dioxide extraction method were explained by Chen et al. This study explained the root cause of apoptosis, which was due to the elicitation of intracellular reactive oxygen species (ROS) generation, eventually caused an increase in in p 53 up regulated modulator of apoptosis (PUMA), Bcl 2 associated X protein (Bax), first apoptosis signal (Fas) and death receptor (DR) 4 expression along with the induction of ERK and c Jun N terminal kinase (JNK) activation. It was observed that the extrinsic apoptotic pathway was initiated by ar-turmerone by activating caspase-3 and caspase-8, which upregulated Fas and DR4. Further, the intrinsic apoptotic pathway was provoked by decreasing B cell lymphoma (Bcl)-2 and Bcl-xL and increasing PUMA and Bax with the release of cytochrome c and activation of caspase-3 and caspase-9 [38] (Figure 3A). Besides this, ar-turmerone possesses profound effect on P388D1lymphocytic leukemia, exhibiting positive rates of Bax and Bcl-2 expression and increased the apoptosis index values. Moreover, the densities of Bax and mRNA increased with no observed changes in the densities of caspase 1, caspase 3, caspase 6, caspase 9, p53, and p21. A marked increase was also observed in T-lymphocytic proliferation and the IL2 production activity increased with increased tumor immunogenicity [39]. Furthermore, the curcumin activities were enhanced in combination with turmerones in human colonic cancer cells (HCT–116, HT–29) and human umbilical vein endothelial cells (HUVEC) [40]. Turmerones also showed promising results when motif–fused by spiropyrrolidine oxindoles through 1, 3 dipolar cyclo–addition events of dienones 2 with azomethine ylides, which were attached to it. These turmerones motif–fused spiropyrrolidine oxindoles showed more potency than cisplatin kept as control over human leukemia cells (K562) and human lung cancer cells (A549) [41].

Even, if significant changes were made to the structures of ar-turmerone, could ameliorate their anticancer effects. Initially, a study explained that the structural changes of ar-turmerone exhibits remarkable anticancer activities by the reduction of size at aromatic ring and their analog formation at the 6-position as observed by a decrease in effective dose-50% value (ED_50_) against lymphocytic leukemia (L1210) cell study [42]. Baik et al. further explained that the orthogonal arrangement between the side chain and aromatic ring of ar-turmerone is responsible for its cytotoxic activities. For this, they considered various substituents on the 1 (or 2) naphthyl group and phenyl ring rather than phenyl group of ar-turmerone and with the help of bioassay compared their cytotoxic activities of these structural analogs in three different cell lines (L1210, HL-60 and K562). The ED_50_ values were observed to be more for HL-60 and K-562 cells than L 1210 which stated that the flat structure of aromatic ring was responsible for its enhanced cytotoxic activities. The reason behind this was attributable to the enlargement of aromatic ring and the restricted rotation of single bond between aromatic ring and C-6 due to the introduction of various substituents at the ortho position of the phenyl ring [43]. Another study identified that changes in morphological characters in ar-turmerone triggered apoptosis in human myeloid leukemia HL-60 cells in a concentration-dependent manner, as it produced internucleosomal DNA fragmentation of approximately 200 bases-pair multiples [44]. Collectively, these studies elucidate that the anticancer activities of turmerones and their isomers on different cell lines trigger cell cycle arrest, apoptosis and their mechanism of action is comparable to curcuminoids. Additionally, the combination of turmerones with curcumin would enhance the bioavailability of curcumin tremendously. Therefore, these studies shape this bioactive component as a novel and potent anticancer tool.

### 4.2. Elemene

Elemenes, (1-methyl-1-vinyl-2, 4-diisopropenyl-cyclohexane) are among the 109 sesquiterpenes present in turmeric [32]. The potency of elemene, isolated from turmeric, has been already stated in China’s food and drug administration and is extensively used in the chemotherapy of the cancer patients in China. Many investigations have led to the fact that elemene can inhibit the tumor growth of various cells, such as ovarian, laryngeal, non-small cell lung, prostrate, melanoma, leukemia, breast, brain, hepatoma, colorectal adenocarcinoma, glioblastoma, and human cervix epithelioid carcinoma cells due to its apoptotic behavior [45]. Hua et al. demonstrated the antitumor activity of elemene by studying the inhibition property of it on HL-60 cell proliferation. This behavior is associated with cell cycle arrest between phase transitions from S to G2M phase, thereby promoting apoptosis [46]. Elemene can also pass through the blood brain barrier combating the carcinomas of the brain due to its small size and lipophilic nature [47]. Elemene is found in three different structural isomers, namely α, β, and γ-elemene; among them, β-elemene is abundantly found and is more active than the other components.

#### 4.2.1. β-Elemene

β-elemene, (1S,2S,4R)-1-ethenyl-1-methyl-2-4-bis(prop-1-en-2yl) cyclohexane, is a sesquiterpene which is studied for its anticancer activity in various cancer cells through its antiproliferative activity with subsequent induction of apoptosis. The research catalog of β-elemene has a range of studies dealing with their effects on different cancer cell lines, mainly on non-small cell lung cancer (NSCLC) cells and the effective mode of action to trigger apoptosis. To begin with, a study explained the effect of β-elemene on these cells caused cell death through the mitochondrial release of the cytochrome c-mediated apoptotic pathway. These lung cancer cells were arrested at the G2-M phase, which caused a decrease in levels of phosphor- cell division cycle (cdc)2 (Thr-161) and cyclin B1, with an increase in the levels of phosphor-cdc2 (Tyr-15) p27kip1 and partially by the check point kinase (CHK)2-dependent mechanism [48]. Li et al. reported that β-elemene sensitizes human NSCLC lines (Aa549 and H460), to cisplatin. This was due to the mitochondria-mediated intrinsic apoptosis pathway which involves IAPs (inhibitor of apoptosis proteins) and Bcl-2 family proteins in a time and dose-dependent manner. Hence β-elemene and cisplatin combination proved to be a promising regimen for the treatment of lung cancer and cisplatin-resistant tumors [49] (Figure 3B). Further, investigation of β-elemene on human non-small cell lung cancer A549 cells by Shu et al. explained that β-elemene inhibited the activity of the P13K/Akt/ mammalian target of rampamycin (mTor)/ribosomal protein S6 kinase beta-1(p70S6K1) signaling pathway, resulting in protective autophagy and apoptosis [50]. Li et al. also reported the rapid inhibition of ERK and Akt activation and upregulation of Casitas B–lineage lymphoma (c-Cb1 and Cb1-b) expression, which enhanced apoptosis in a dose-dependent manner in lung cancer cells (A549) [51]. Furthermore, the intensive antiproliferative effect of β-elemene on NSCLC cells was proved by AMP-activated protein kinase (AMPKa) and ERK1/2 mediated inhibition of transcription factor Sp1, followed by reduction in DNA (cytosine-5)-methyltransferase 1 (DNMT1) protein expression. This study also found that metformin intensifies the effect of β-elemene by blocking Akt signaling and inhibits DNMTI protein expression. In addition, the reciprocal of AMPKa and ERK1/2 signaling pathways are responsible for β-elemene responses [52]. It was also noticed that β-elemene, isolated from *C. aromatica* and combined with etoposide, proved very beneficial for lung cancer [53].

β-elemene has a promising therapeutic role in human SGC7901 and MKN45 gastric cancer cells. β-elemene inhibited the cell viability and clonogenic survival of gastric cancer cell that were decreased in a dose-dependent manner, with induction of apoptosis [54]. The effects of β-elemene in murine hepatocellular cancer, and the relation with its expression level of c-Met (a receptor tyrosine kinase) were examined over neoplastic tissues. Analysis through western blot, immunofluorescence analysis and reverse transcription polymerase chain reaction (RT-PCR) confirmed the presence of c-Met expression in H22 hepatocellular carcinoma xenograft model in vitro and in vivo. The test results showed that the induction of growth inhibition in murine hepatocellular carcinoma cells by β-elemene was brought about by downregulation of c-Met expression [55]. It was further found by Zhan et al. that β-elemene inhibits human renal carcinoma (RCC) 786-0 cell proliferation by inhibition of PI3K/Akt/mTOR and mitogen activated protein kinase (MAPK)/ERK signaling pathways, thus inducing protective autophagy and apoptosis [56]. Another study explored the efficacy of β-elemene when treated with taxanes over ovarian cell lines A2780/CP70 and its parental cell lines A2780. The results suggested that this combination reduced cell viability, arresting cell cycle at G2/M phase, increasing cell apoptosis [57]. Zhang et al. studied that β-elemene has the potency to reverse tumor MDR (multi-drug resistance) and revealed that their combination with doxorubicin enhanced the anticancer activity of the latter due to the presence of β-elemene. This was observed by the increase in the intracellular accumulation of rhodamine 123 and doxorubicin in both SGC7901/ADR cells and K562/DNR cells which influenced the inhibition of p-glycoprotein (p-gp). β-elemene downregulated the phosphorylation of Akt pathway and upregulated E3 ubiquitin ligases, Cb1-b and c-Cb1 when treated on SGC7901/ADR cells. Thus, β-elemene could target gastric cancer cells and p-gp overexpressing leukemia to increase the efficacy of doxorubicin treatment [58].

In this series, β-elemene was found to be influential in glioblastoma therapy by activating p38 MAPK, which is an important factor for the antiproliferation effect [59]. It has potential effects on glioblastoma multiform (GBM), which is associated with tumor, poor radiotherapy prognosis and resistance to temozolomide (TMZ). Subsequent investigation of β-elemene in radio sensitivity and chemo sensitivity of GBM cells lead to the conclusion that it can be combined with radiotherapy and TMZ. In this way, assist in blocking the survival and proliferation of the cell lines of GBM, through the inhibition of DNA damage repair. Additionally, a decrease in phosphorylation of Ataxia telangiectasia mutated (ATM), ERK and Akt were observed when these cells were treated with β-elemene, followed by chemotherapy and radiotherapy. Thus, β-elemene is beneficial in combination therapy on GBM cells [60]. Furthermore, Zhu et al. with a background of previous accounts on the role of β-elemene in arresting glioblastoma cells (U87 and C6) in G0/G 1 phase, showed that by regulating the glia mutation factor β/mitogen activated protein kinase 3/6/p38 and extracellular signal regulated kinase 1/2/B cell lymphoma 2/surviving pathways, inhibition of cell proliferation was facilitated and promotion of in vitro and in vivo differentiation of glioblastoma stem cells (GSCs). They found that this was a crucial step in the treatment of glioblastoma. For this, they recognized many GSC markers that can be used for the differentiation of these tumor cells such as human CD133, ATP-binding cassette subfamily G member 2 (ABCG2), glial fibrillary acidic protein (GFAP), Notch 1, and Sonic hedgehog (SHH). These findings led their investigation to understand the effects and mechanism of β-elemene on cell survival, stemness differentiation, proliferation, and epithelial mesenchymal transition (EMT) in vitro and in vivo. Along with this, the downregulation of expression levels of stemness markers CD133, ATP-binding cassette subfamily G member 2, mesenchymal markers N-cadherin, and β-catenin were facilitated. Moreover, upregulation of expression levels of differentiation related to effectors of glial fibrillary acidic protein, sonic hedgehog, Notch 1 and epithelial marker (e-cadherin), in vitro and in vivo, were promoted. In addition, their studies underlined that β-elemene promoted apoptosis, impaired invasiveness in glioblastoma cells and suppressed growth of animal xenografts and inhibit proliferation. Furthermore, it showed an increase in expression level of vimentin in vitro with noticeable inhibition of β-catenin that enhanced the effects of EMT-inhibition, antiproliferation, and specific marker expression regulatory of β-elemene [61].

Apart from the above stated apoptotic pathways, β-elemene is thought to modulate through various signaling pathways to decrease the antiapoptotic protein expression of HIF-1 α, vascular endothelial growth factor (VEGF), Bcl-2, Survivan, cellular FLICE- like inhibitory protein (cFLIP), upregulating apoptotic proteins mitogen activated protein kinase kinase (MKK)3, MKK6, P38 MAPK, and suppress the mTOR pathway [12]. They also works in combination with other anticancer drugs, like tamoxifen, arsenic trioxide, gefitinib, etoposide, and cisplatin to give results that are more positive. It has shown its efficacy in overcoming cisplatin-resistant (A2780/CP) and the cisplatin-sensitive (A2780) ovarian cancerous cells. In the same way, it partially reverses Adriamycin-resistant breast cancer cells (MCF-7/ADM) [12].

Moreover, there were many studies that indicated the efficacy of the derivatives of β-elemene as an anticancer agent. Xu et al. synthesized and characterized fourteen β-elemene derivatives containing morpholine, piperzine, terahydropyrrole, cyclohexamine, or thiophenylethylamine groups. These derivatives were compared with β-elemene for their proliferative activity in human leukemia K562, gastric carcinoma SGC-7901, cervix epithelioid carcinoma HeLa cells. This proved that 13,14-bis(cis-3,5-dimethyl-1-piperazinyl)-β-elemene and 13,14-bis(cyclohexamino)-β-elemene have similar potency as β-elemene [62]. Even, β-elemene mono-substituted amine, rhenium and ether coordinated complex structure exhibited in vitro antiproliferative activity in human cervix epithelioid carcinoma HeLa cells. This reduction of cyclin D1 protein expression and Rb phosphorylation seized the cell cycle at G1 phase [63]. Moreover, three synthetic analogs of β-elemene, such as β-elemenal, β-elemene fluoride, and β-elemenol have also displayed significant anticancer activities by the considerable suppression in the non-small cell lung cancer cell proliferation. Among them, β-elemene enhanced cisplatin activity by causing lung cancer cell death, which was regulated by the CHK2-mediated cdc25C/cdc2/cyclin B1 signaling pathway to block cell cycle progression at G2/M phase [64]. Among the three analogs of β-elemene, namely, Lr-1: [(R or S)-2-((1R,3S,4S)-3-isopropenyl-4-methyl-4-vinylcyclohexyl)-propane-1,2-diol], Lr-2: [(S)-2-((1R,3S,4S)-3-isopropenyl-4-methyl-4-vinyl-cyclohexyl)-propane-1,2-diol, and (R)-2-((1R,3S,4S)-3-isopropenyl-4-methyl-4-vinylcyclohexyl)-propane-1,2-diol] and Lr-3 [1-((1R,3S,4S)-3-isopropenyl-4-methyl-4-vinyl-cyclohexyl)-ethanone], Lr-1 and Lr-2 can be used as an alternative to β-elemene for the treatment of brain tumors. For this, the cytotoxic efficacy of β-elemene and its synthetic analogs (Lr1 and Lr 2) in the brain tumor cell lines U-87MG, CCF-STTG1, and A172 were compared. It was observed that β-elemene suppressed tumor cell survival and mediated induction of apoptosis via signaling pathways. This study provided evidence that the synthetic analogs of β-elemene are also therapeutically potent compounds [65]. Another derivative of β-elemene, named *N*-(β-elemene-13-yl) tryptophan methyl ester (ETME), when combined with arsenic trioxide (ATO), synergistically enhances the antiproliferative activity and apoptosis of hepatocellular carcinoma (HCC). It also downregulated Bcl-2 and pro-proteins of caspase family, decreased the mitochondrial membrane potential (MMP), and facilitated upregulation of BH3-interacting domain death agonist (Bid) and Bax, which were reversed by pifithrin-α, p53 inhibitor. Hence, reducing the tumor weight and volume in nude mice, MMP in HCCSMMC-7721 cells, and brought cell cycle arrest at the G2/M phase [66]. Chen et al. studied that novel furoxan-based NO-donating β-elemene hybrids are promising anticancer agent [67]. Their studies also demonstrated isopropanolamine derivatives of β-elemene are effective chemotherapeutic agents [68].

Moreover, Jiang et al. reviewed and explained that the upregulation of pro-apoptotic signals and downregulation of anti-apoptotic signals. They concluded that this could be the possible origin of the mechanism of action of β-elemene in various cancerous cells such as melanoma cells, glioblastoma cells, leukemia, hepatocellular carcinoma cells, human bladder cells, thyroid cells, ovarian cancer cells, and prostrate cells [69]. Recently, a research demonstrated that β-elemene can be a promising tool in doxorubicin resistant breast cancer cells by altering the functionality and quantity of D-luciferin potassium salt, an ATP-binding substrate cassette transporter also known as ABC transporters. The researchers used bioluminescence imaging for studying the efflux kinetics of ABC transporters, which showed deadening functionality. The colorimetric test for assessing cell metabolic activity (3-(4,5-dimethylthiazol-2-yl)-2,5-diphenyltetrazolium bromide) tetrazolium reduction assay (MTT assay) and lower IC_50_ values explained the increased effect of doxorubicin on doxorubicin resistant cells when combined with β-elemene [70]. Guo et al. explained the mechanism of action and the mode of interaction with ABCB1 expression of β-elemene by molecular docking analysis. Their study analyzed that β-elemene interacted hydrophobically with the binding pocket of ABCB1 substrate, as well as its most feasible docking site-carrying double bond, connected via π-π interaction with aromatic residues to increase ABCB1 protein sensitivity. Thus, the possible mode of anticancer activity of β-elemene in altering MDR through the inhibition of ABCB1 transporter efflux activity was determined [71]. Similar docking studies have established the potent biological activities of sesquiterpenes from essential oils [72]. But more molecular docking studies should be performed to authenticate its therapeutic potential. These studies prove that β-elemene is another major compound of non-curcuminoids possessing anti-cancer activities. It can alter and inhibit cancer cells of various types, such as brain, lung, the gastric system, and so on. In addition to this, their derivatives also possess potential anticancer activities as seen through these literatures. It is commendable that a strong component, such as β-elemene, possesses influential biological activities when taken alone or in combination to improve most chronic diseases like cancer and revamp multidrug-resistant cancers.

#### 4.2.2. δ-Elemene

δ-Elemene is also a terpene, which is the structural isomer of β-elemene with three consecutive isoprene units. It is reported that their anticancer activities are initiated by activation of caspase signaling pathway. This initiation led to the cleavage of poly (ADP-ribose) polymerase (PARP) colorectal adenocarcinoma and Hela cells along with the activation of caspase-3. It also exhibited promising behavior when treated for NCL-H292 lung cancer cells, as it showed apoptotic behavior in Hela cells by attenuating L-glutathione or the Z-D (OM e) E (OMe)VD(OMe) fluromethylketone (z-DEVD-fmk) increment of p38 MARK, and showed inducible nitric oxide synthase levels [73]. These studies prove that even the isomers of elemene exhibit anticancer activities when examined on different cancerous cell lines. The rationale behind this is apoptosis and cell cycle arrest, which are similar to curcuminoids. Thus, these biological components can be considered along with curcumin or alone, as they have meager side effects, and has very promising efficiency to retaliate against cancer.

### 4.3. Furanodiene

Furanodiene, (5E,9E)-3,6,10-trimethyl-4,7,8,11-tetrahydrocyclodeca[b]furan, another sesquiterpene extracted from the volatile oil fraction of turmeric by CO_2_ supercritical fluid technique. Fuanodiene had inhibitory effect on various cells, such as HL-60 (promyelocytic leukemia), HeLa (cervical carcinoma), SGC—7901 (gastric cancer) HeLa, K562 (leukemia), A549 (lung adenocarcinoma), MDA-MB-435s (breast cancer) HT-1080 (fleshy tumor), Hep-2 (laryngocarcinoma), SMMC-7721(hepatoma), and PC3 (prostatic carcinoma), displaying their benevolent potential with varied mechanisms of action. In hepatoma cells (Hep G2), the potency of furanodiene was demonstrated by DNA fragmentation assay. The inhibition of cell proliferation brought cell cycle arrest at the G2/M phase and apoptosis through the mitochondria-caspase apoptotic pathway, which activated p38, and inhibited the ERK/MAPK signaling pathway [74]. Another study evaluated the effects of furanodiene on human leukemia cell HL60 by DNA fragmentation and indicated that furanodiene brought apoptosis through cleavage of caspase 3, caspase 8, caspase 9, and PARP. Along with this, the Bcl-2 family proteins and BID protein were activated, whereas Bax, Bcl-xl, and Bcl-2 proteins were not at all affected by the stimulation of furanodiene. It was also seen that furanodiene promoted upregulation of tumor necrosis factor receptor 1 (TNFR1), with the formation of TNFR1 complex along with the production of TNF-α in HL60 cells [75]. Ba et al. studied the anticancer activity of furanodiene on HeLa, HL-60, U251, and Hep-2 cells. and explained that furanodiene is effective against uterine cervix cancer (U14) cells and has a protective effect on immune function [76]. Further, the effect of furanodiene, studied on HeLa cells, revealed that furanodiene in a dose-dependent manner caused cell death. This proved its efficacy on sarcoma 180 cells in mice and uterine cervical cancer growth [77]. Further, successful development and validation of the pharmacokinetic parameters of furanodiene in rat plasma through tandem mass spectrometry and liquid chromatography were studied. The results explained that the bioavailability of furanodiene in rats was about 49%, which proved that their method of analysis might be helpful to prove the potency of furanodiene as an anticancer agent [78]. Another study showed that furanodiene inhibited protein expression of total cyclin D1, p-cyclin D1, p- cyclin-dependent kinases inhibitors (CDK)2, total CDK2, pRb, total Rb, Bcl-Xl, and Akt. These expressions suppressed breast cancer cell growth both in vitro and in vivo and increased the protein expressions of Bax and Bad, along with elevation of proteolytic cleavage of caspase 9, as well as caspase 7 and PARP [79]. Furanodiene has also proved its efficacy in the inhibition of human umbilical vascular endothelial cells and proliferation carried out by VEGF [80].

There were many studies, which reflected the usefulness of furanodiene in combinational therapy to combat various cancer cells. Xu et al. studied the antiproliferation activity of furanodiene on lung cancer cells A549, NIH-H1299, and 95-D and studied its usefulness in combination therapy with paclitaxel. This study observed the inhibition of colony formation in 95-D cells and A549 cells and that it facilitated upregulation of protein expression levels of binding immunoglobulin (BIP), mRNA and C/EBP homologous protein (CHOP), as well as its accumulation in the nucleus, inducing endoplasmic reticulum (ER) stress. Further, they studied that furanodiene efficiently causes cell metastasis in 95-D lung cancer cell, by activating the signaling molecules and regulating G1 cell cycle arrest, thereafter inducing apoptosis and autophagy. This autophagy was involved in the enhancement of the expression of light chain3-II (LC3-II) in the protein level. Due to this, downregulation of the protein levels of full PARP surviving, Bcl-2 and pro-caspase 7 and upregulation of cleaved PARP, affected the signaling molecules that promote apoptosis. It was also seen that furanodiene inhibited cell proliferation in a concentration-dependent manner as well as caused blockage of cell cycle progression in G1phase by downregulating the protein levels of cyclin D1 and CDK6 and upregulating those of p27 and p21 in 95-D cells [81]. Zhong et al. also studied the combined effects of tamoxifen (TAM) and furanodiene in human breast cancer cell in vitro. This research showed that the enhancement of growth inhibitory and pro-apoptosis behavior of furanodiene, enhanced the pro-apoptosis and growth inhibitory activity of tamoxifen. This activity induced cell cycle arrest via mitochondrial-caspase dependent, PARPg-independent and CDKs-cyclin signaling pathway in human breast cancer cells without affecting the cytotoxicity of Tamoxifen [82]. Furanodiene, when treated on breast cancer cell lines MCF-7 and MDA-MB-231 suppressed proliferation and increased the lactate dehydrogenase (LDH) release in a dose-dependent manner. It also depolarized mitochondrial membrane potential, chromatin condensation, and DNA fragmentation bringing cell cycle arrest at G0/G1 phase. Furanodiene, when injected intraperitoneally in mice, witnessed tumor suppression in a dose-dependent manner in vivo [83]. Zhong et al. research showed that furanodiene is a promising agent for treating chemo resistant breast cancer cell. They studied that when doxorubicin-resistant breast cancer cells (MCF-7/DOXR) are treated with furanodiene, showed an alteration in mitochondrial function as well as the levels of ATP are reduced causing apoptotic cell death. In these cells, the phosphorylation of AMPK and AMPK pathway, ATP citrate luase (ACLY), 3-glycogen synthase kinase (GSK)–3β and AICAR (5-aminoimidazole-4-carboxamide-1-β-4-ribofuranoside, acadesine), an AMPK activator, augmented by furanodiene by induction of anticancer activity [84] (Figure 3C). Further, a study explained the combination therapy with furanodiene and doxorubicin, by inference of cell migration and cell invasion, when taken in a non-toxic concentration, in highly metastatic breast cancer MDA-MB-231 cell in vitro. This combination showed downregulation of integrin αV, and β-catenin expression, and inhibited the phosphorylation of paxillin, FAC (focal adhesion kinase), p85, Akt and decreased the MMP-9. Additionally, it reflected no influence on the expression levels of neural Wiskott-Aldrich syndrome protein (N-WASP), cdc42, RhoA and α/β tubulin. Thereby, the effects of doxorubicin on the tubulin cytoskeleton remained unaltered. Hence, proved that furanodiene is highly beneficial when combined with doxorubicin on cancerous breast cells [85]. Hence, the advantage of furanodiene is that it can be used alone or in combination for different types of cancer and drug resistant cancers. These extensive studies provide a pathway for furanodiene to be established as a therapeutically effective natural compound for cancer treatment.

### 4.4. Furanodienone

Furanodienone is a sesquiterpene with a structure characterized by a cyclodecane ring, substituted with two methyl groups and an isopropyl group. It is also considered as a promising anticancer agent. The influence of furanodienone on human breast (MCF-7, MDA-MBA-231, and T47D) cells was investigated by Li et al. Their studies showed marked decrease in cell growth in a dose-dependent manner, by inhibiting estrogen receptor α signals and mRNA expression levels without effecting ER beta. It also blocked 17β-estraiol (E2)-stimulated MCF-7 cell proliferation and E2-stimulated estrogen response element driven plasmid activity and removed E2 targeted gene expression (Cyclin D1, Bcl-2, and c-myelocytomatosis (MYC)) which inhibited cell proliferation with induction of apoptosis [86]. Furanodienone also inhibited signaling pathway of epithelial growth factor (EGFR)/human epithelial growth factor receptor (HER2) of HER 2-overexpression of human breast cells (BT474 and SKBR3) by G1 cell cycle arrest and apoptosis [87]. Recently, Jiang et al. explained that when colorectal carcinoma cells were treated with furanodinone, it induced apoptosis with the help of reactive oxygen species. Furanodinone showed an upregulation of p21cip1 which caused decrease in cyclin D1, cyclin E, cdk4/6, and cdk 2 and this ultimately resulted cell cycle arrest at G0/G1 phase. In addition to this, the activation of ROS was enhanced by N-acetyl cysteine (NAC), increasing the phosphorylation of JNK, p38, and decreased ERK. This is further explained by caspase-dependent intrinsic and extrinsic mitochondrial pathway, stimulated by MAPK which facilitated the reduction of Bcl-2 and increased Bax with induction apoptosis [88] (Figure 3d). Therefore, the outcome of these studies conducted on breast and colorectal cancers suggests that furanodinone is an effective anticancer agent.

### 4.5. Curcumol

Curcumol, (1S,2S,5S,9S)-9-isopropyl-2-methyl-6-methylene-11-oxatricyclo (6.2.1.01,5) undecan-8-ol, is a potent anticancer sesquiterpenoid that inhibits proliferation of Hela, OV-UL-2, MCF-7, and MM231 cancer cells by RNA synthesis in a concentration-dependent manner. It caused cell death in ARTE- induced human lung adenocarcinoma (ASTC-a-1) with the induction of cell cycle arrest at G2/M phase with nuclear fragmentation, phosphatidylserine externalization, and rapid Bax translocation from the cytosol into mitochondria [89]. Huang et al. revealed that curcumol brought apoptosis in nasopharyngeal carcinoma CNE-2 cells by downregulation of NF-κB. This compound also showed promising anticancer effects in ASTC-a-1 cell by apoptosis mechanism, mediated by caspase-independent mitochondrial pathways [90]. Curcumol when treated on hepatic stellate cells (HSC) induced apoptosis by suppression of PI3K, Akt and Ikappa B. This suppression was due to the decrease in NF-κB and its DNA binding activity leading to the decrease in Bcl-2, Bcl-xl, and increase in Bax expression along with the increase in caspase 3 [91] (Figure 3e). In addition, the anticancer effects on colorectal cancer LOVO cells were enhanced by curcumol. This study showed that when curcumol was treated on these cells decreased Bcl-2 and increased Bax to induce apoptosis. Further, the suppression of insulin-like factor-1 receptor (IGF-1R) escalated the phosphorylation of p38 MAPK and decreased Camp-response element (CREB) expression to trigger apoptosis [92]. Thus, these studies elucidated the anticancer activities of curcumol on different types of cancers through different mechanism of action that ultimately led to cell cycle arrest and apoptosis that hold a promising path to acknowledge them as effective anticancer agents.

### 4.6. Cyclocurcumin

Cyclocurcumin,(E)-2-4-hydroxy-3-methoxyphenyl-6-(4-hydrox-3-methoxystyryl)2H-pyra-4(3H)-one, isolated and identified from turmeric have some structural similarities to curcumin. They are formed by the addition of enol oxygen group through intramolecular Michael addition to enone group [9]. They have shown inhibitory action on breast cancer (MCF-7) cells [12]. Simon et al. investigated that cyclocurcumin acts as a nematocidal agent when combined with curcumin [93]. Moreover, studies revealed that cyclocurcumin exhibited light induced trans-cis isomerization [94]. Even, the molecular docking study provided an in-depth mechanism by which ternary DNA topoisomerase I and II complexes are induced by the derivatives of curcumin particularly cyclocurcumin and curcumin sulphate. This factor was determined by free energy binding analysis and polar- hydrophobic interactions. These topoisomerases are enzymes which resolve the topological problem such as transcription, recombination, chromatin remodeling, replication that are responsible for causing cancer. Docking of cyclocurcumin and curcumin sulphate were found to be the most promising, as their binding modes were at the DNA cleavage site which corresponded to the DNA base pairing axis, equivalent to the topoisomerase I and II inhibitors. Thus, these docked complexes are converted into permanent DNA strand breaks by cellular processes inducing apoptosis. This docking study provided an insight on the efficiency of cyclocurcumin as therapeutically potential compound for treating various cancers such as ovarian, brain, lymphomas (Hodgkin and non-Hodgkin), lungs, and adrenocortical cancers [95]. Although, these compounds fall under non-curcuminoids, they exhibit certain structural similarities to curcumin. Further, their potential anticancer activities are highlighted by the successful molecular docking study.

### 4.7. Calebin A

Calebin A(CA),(3E)-4-(4-hydroxy-3-methoxyphenyl)-2-oxobut-3-en-1-yl(2E)-3-(4 hydroxy-3-methoxyphenyl) prop-2-enoate), isolated from *C. longa*, has a ferulic acid ester bond which lacks the 1, 3-diketonic structure of curcuminoid compounds. Many researchers have shown that calebin A is beneficial in combating numerous cancerous cells such as colon, gastric, multiple myeloma, breast cells as well as multi-resistant cancer cells. A study of calebin A suggested that it is an effective compound in chemotherapy for multi-drug resistant cancers, as it inhibits the growth of the cell and induced apoptosis in SGC7901/Vincristine cells, a multi-resistant (MDR) human gastric adenocarcinoma cell line, A549/DDP (cisplatin)-MDR pulmonary carcinoma cell line and HepG2/ADM (Adriamycin)-MDR hepatoma cell line. This study revealed that drug efflux function of p-glycoprotein was inhibited with their treatment, but the expression level of P-glycoprotein remained the same. Calebin A was also found to modulate the activities of MAPK, which included increased protein kinase of p38, 38kda activity. They also, decreased JNK as well as ERK. These results suggested that this is a genuine compound for the treatment of human gastric and other MDR cancers [96]. Calebin A was found to be very effective for HCT-116 type colon cancer cells and has proved to be more potent, when compared with curcumin. The expression of regulatory protein of cell cycle such as cyclin-A, cyclin-B, cdc25A, cdc2 levels are decreased by calebin A, causing S and G2/M cycle arrest and increases the CDKI p53 and p21. In fact, this compound increases the ROS levels, which again leads to cell cycle arrest as it promotes the DNA damage response and upregulate the phosphorylation of H2A histone family member X on serine 139 to form (γH2AX ), CHK1, and CHK2. When intraperitoneally administrated on colon tumor cell in vivo, they showed decreases in the tumor volumes, tumor sizes and the cell nuclear antigen (PCNA) protein levels [97] (Figure 3F). Tyagi et al. identified calebin A from *C. longa*, utilized mouse macrophages and electrophoretic mobility shift assay in vitro. This study showed that CA causes suppression of receptor activator of nuclear factor kappa-B ligand (RANKL)-induced osteoclast differentiation of macrophages and forms osteoclasts, with downregulation of marker gene expression of RANKL-induced osteoclastogenesis, including calcitonin receptor (CTR), tartrate-resistant acid phosphatase (TRAP), nuclear factor of activated T-cells cytoplasmic (NFATc–1), and cathepsin K. Additionally, suppression of phosphorylation, followed by the degradation of kB, NF-κB, which were the main source for the suppression of osteoclastogenesis that induced multiple myeloma and breast cancer [98]. Taken together, calebin A, alike curcumin exerts anticancer activities mainly on colon cancer cells through induction of apoptosis and cell cycle arrest. These compounds have positive results on breast cancer cells and even on multi-resistant cancers.

### 4.8. Germacrone

Germacrone, (3E, 7E)-3, 7-dimehyl-10-propan-2-ylidenecyclodeca-3, 7-dien-1-one, a well-known anticancer terpenoid, were isolated from Curcuma Rhizomes. Germacrone, through cell cycle arrest and induction of apoptosis, brought about proliferation in breast cancer cell in vitro. This demonstrated marked rise in LDH release and induction of depolarization of mitochondrial membrane potential in breast cancer cells in a dose-dependent manner [99]. Bin et al. explained that germacrone has proved to be beneficial as antitumor agent on human hepatoma cell lines (HepG2 and Bel 7402), by activating CDK1, inducing P21 and reducing the protein expression of cyclin B1. Germacrone also showed oxidative damage in human hepatoma cells along with the increase of the intercellular reactive oxygen species. Various tests performed such as flow cytometry, MTT assay and fluorescent microscopy concluded that germacrone exhibited antiproliferative effect on Bel7402 and HepG2 cells. Through annexin V/P staining and Hoechst 33258, it was observed that in a dose-dependent manner, and amplification in the downregulation of Bcl-2/Bcl-xl and upregulation of Bax were observed. This amplification was associated with cell apoptosis, G2/M phase cell cycle arrest and caused reduction in the cyclin B1 and its activating partner CDK1, which induced p21, along with the upregulation of p53 and ROS. Thus, these mechanisms proved germacrone as potent anticancer agent for liver cancer [100] (Figure 3G). The connectivity between germacrone induced apoptosis in HepG2 cells and Janus activated kinases (JAK2)/signal transducer (STAT3) signaling pathway facilitated anticancer activities. This analysis explained that germacrone downregulated the activation of JAK2/STAT3, regulated Bcl-2 family member and p-53, which are STAT regulated, to bring about apoptosis inHepG2 cells [101]. Anticancer activity of germacrone on breast cancer cells was explained by Xie et al. They explained the aftermath of germacrone on human breast cancer MCF-7/Adriamycin (ADR) MDR cells with the help of flow cyclometry. The results revealed that germacrone in a dose-dependent manner brought about apoptosis and inhibited MDR1 gene promoter by reducing the p-glycoprotein expression. The combination of germacrone with ADR increased apoptosis, resulting in the antiapoptotic protein Bcl-2 expression levels and refinement of pro-apoptotic proteins Bax and p53 expression levels in MCF-7/ADR cells, which was much low when compared to ADR treatment alone [102]. Another research showed the effectiveness of germacrone in combination therapy with other drugs, namely 5-fluorouracil and methotrexate, in elevating its antitumor activity, on positive breast cancer cells estrogen receptor α (ER-α). These signals at transcriptional level undergoes inhibition in MCF-7 cells and replace it to estrogen response element on chromatin. Thus, with the inhibition of accessibility of estrogen-induced chromatin, target gene promoter, compromised with the binding of RNA polymerase II and complex of sucrose/switch non-fermentable chromatin remodeling, caused cell-proliferation [103]. Moreover, the derivatives of germacrone had inhibitory effects on Bel-7402, HepG2, A549, and HeLa cells. These derivatives had stronger inhibitory effects on c-Met kinase, which have been explicated in many cancers [104]. A molecular docking study performed for essential oils against bacterial proteins present in enzymes 2X5O, 3UDI, murD ligases and 3TYE. These proteins were subjected to docking as co-crystal ligands and the docking score was validated by monitoring the docking of individual components of essential oil to the active sites of protein. This study highlighted that germacrone proved highly promising among the other constituents with a high grade of interaction with proteins. Hence, through this study, germacrone cleared a definite pathway, in possessing enormous biological activities. However, more studies should be conducted for germacrone from *C. longa* to have an in-depth understanding of this moiety [105]. Therefore, these components are influential as anti-cancer agent as they are least harmful for human health. Hence, it is evident from these studies that germacrone exhibited anticancer activities through cell cycle arrest and apoptosis which is comparable to the proven mechanism of curcumin [13].

### 4.9. Bisacurone

Bisacurone, (6S)-6- [(1R, 4S, 5S)-4, 5-dihydroxy-4-methylcyclohex-2-en-1-yl]-2-methylhept-2-en-4-one, is a terpenoid, known as a very potent anticancer agent. Studies showed that vascular cell adhesion molecules-1 (VCAM-1) promoted carcinogenesis. Therefore, Sun et al. carried out investigation to find an herbal remedy for its suppression and found that Bisacurone in TNF-α activated by human umbilical vein endothelial cells (HUVECs) in a dose-dependent manner inhibits the level of VCAM-1. It showed significant blockage of the NF-κB p65 carried to the nucleus, phosphorylation of inhibitory factor kBα and suppression of ROS. Bisacurone, also showed inhibition of phosphorylation of protein kinase C (PKC) and Akt that helps in the regulation of VCAM-1 by TNF-α. Thus, they prove to be potential anticancer agent by their promising results in regulating VCAM-1 [106] (Figure 3h).

### 4.10. Curdione

Curdione, is a monoterpene isolated from the essential oils of turmeric and has been clinically used for the treatment of cervical cancer in China. Prostaglandins are known to promote carcinogenic processes and curdione has been found exorbitantly efficient enough to inhibit prostaglandin biosynthetic enzyme cyclooxygenase. This facilitated the repression of COX-2 mRNA expression and inhibition of the fabrication of prostaglandin (PG)-E2 in the lipopolysaccharide (LPS) stimulated (RAW-264.7) mouse macrophages in a dose-dependent manner [107]. Kong et al. studied MCF-7 and MDA-MB-231 breast cancer cell proliferation both alone, as well as in combination to find that an unpredictable intra herb interaction exist that should be considered while formulating curdione, germacrone, and furanodiene, isolated from *C. wenyujin*. In addition, germacrone and curdione showed no antiproliferative activity while furanodiene reacted oppositely, which were identified with the changes in mitochondrial membrane and morphological changes. Moreover, germacrone enhanced antiproliferative effect of furanodiene. Curdione showed no effect on antiproliferative effect of furanodiene, however, had some combined effects on the antiproliferative effect of germacrone and furanodiene [108]. Further, the inhibition of breast cancer cell proliferation in nude xenograft mouse elucidated the anticancer effect of curdione in a dose-dependent manner. This study utilized various tests like Jc-I assay, MTT, flow cyclometry, and Western blot and analyzed the cleavage of protein like caspase3 and caspase 9, followed by the increase in Bax and impairment of mitochondrial membrane potential, that brought about apoptosis in a concentration-dependent manner. These observations concluded that curdione induces apoptosis and is an effective chemotherapeutic agent [109] (Figure 3I). Li et al. successfully validated the pharmacokinetic parameters of curdione and curcumol from *C. longa* extracts in rat’s blood and liver by micro-dialysis coupled with rapid resolution tandem mass spectrometry and liquid chromatography (RRLC). Thus, this pharmacokinetic study provided a promising and useful platform for future studies related to its application in anticancer and anti-inflammatory activities [110]. Hence, these studies suggest that curdione give promising results when taken with other non-curcuminoids. Additionally, they work excellently on breast cancer cells, which provide knowledge on their predominant anticancer potential.

## 5. Critical Analysis of the In-Vivo Effects of Non-Curcuminoids

There are many studies, which prove the efficiency of non-curcuminoids. A recent study demonstrated that the total extract of turmeric is more superior to curcuminoids alone. This fact was assessed by methylthiazolyldiphenyl tetrazolium bromide (MTT assay), which concluded that the cell viability or proliferation decreased in cancer cells especially lung cancer cells. Additionally, isobolographic assessment revealed that when total extract of turmeric when used synergistically with cisplatin, the dose of cisplatin used can be reduced than the actual dose to bring similar effect with less toxicity and adverse effect as treatment with cisplatin alone [111]. Many evidence-based studies have also been initiated since long, which emphasize the therapeutic potential of the components of turmeric. Numerous clinical trials have been conducted for curcumin, which denote the clinical potential of them having a safe uptake of 12 g/day. These clinical studies also claim that curcumin is very efficient in treating various forms of cancer such as colorectal, breast, head, and neck, pancreatic, and so on [112]. Even clinical trials on the safety and therapeutic efficacy of curcumin-free compounds have been reported, some of which are still ongoing [113]. Among them, β-elemene, a green drug extracted from *C. longa*, which is still extensively studied and also accredited by the Ministry of Health People’s Republic of China and State Pharmacy of China to be the most beneficial compound for medical purposes, mainly against numerous forms of cancer including non-small cell lung cancer cell (NSCLC), liver, brain, esophageal squamous cell carcinoma, and so on [114]. In earlier trials, the safety and efficacy of β-elemene used in combination was validated through controlled, randomized clinical trials. These trials involved around 1467 patients divided into group of two for studying NSCLC and small cell lung cancer (SCLC). Accelerated improvement in the survival and tumor response rate were observed with minimal side effects in patients treated with β-elemene injection as adjunctive for lung cancer in contrast to patient given medication without β-elemene injection [115]. Joshi et al. also validated that turmeric oil is safe as per the phase I trials on nine healthy volunteers and efficient as a chemopreventive agent especially in submucous fibrosis [116]. Another successful investigation was carried on 102 patients with esophageal squamous cell carcinoma who underwent chemoradiotherapy and, thereafter, were subjected to surgery. The recurrence rate with usual chemotherapy regimen for esophageal squamous cell carcinoma was about 60%, decreasing the survival rate. Therefore, this clinical trial with β-elemene treatment increased the survival (overall and progression free) rate and decreased the toxicity level related to synchronous chemoradiotherapy with cisplatin and 5-fluorouracil regimen [114].

Certain ongoing clinical trials are facing efficacy problems due to lack of promised delivery at specific tissue sites, these can be rectified if non-curcuminoids are encapsulated in different vesicles to facilitate target delivery. The combination of turmeric oleoresin (mainly ar-turmerone and 1,8 cineole) with β-cyclodextrin through co-precipitation or kneading method can form inclusion complexes, improved the solubility of turmeric oleoresin, which was determined through phase solubility studies. These complexes can improve the biological activities of non-curcuminoids significantly [117]. In this way, there are numerous studies, which improve the authentic properties of non-curcuminoids specially the solubility, bioavailability as well as stability and focus on such formulations can be a breakthrough in the clinical trials. Our research team, Aurea Biolabs, took initiative and formulated a novel technique, PNS technology, involving a complete natural turmeric matrix comprising of turmeric water extract, turmeric essential oils, and curcuminoids. In addition, a series of clinical trials were conducted to prove the potency of this matrix to deliver efficiently the most bioavailable form of curcumin to the target site. An open labeled parallel arm comparative study with two other curcumin formulation was conducted. This study engaged 45 healthy male candidates who were given a single dose of 500 mg through oral administration. This comparative study proved the worth of this formulation in contrast to the other two formulations in improving the solubility and bioavailability enormously. To ascertain the claimed high bioavailability of this formulation, a pilot crossover study was undertaken on 12 healthy male adults and the blood samples for curcumin content were analyzed. The results were promising as 10 times more increase in bioavailability was witnessed than curcumin (95% purity) as measured by concentration maximum (C_max_) and area under the curve (AUC) values [118]. With the continuous success of these studies, another clinical study was carried as a double-blind, randomized, two-dose, placebo-controlled and three-arm parallel study with 36 rheumatoid arthritis patients to examine the efficacy and safety of highly bioavailable form of curcumin in turmeric matrix fabricated by Aurea Biolabs. Both 250 mg and 500 mg doses of this novel formulation in this study were well tolerated and devoid of adverse effects [119]. The outcomes of these concurrent studies are promising and help to evaluate that β-elemene is a multifaceted compound which could be significantly beneficial in chemotherapy when treated in combination with other regular supportive medicaments for various cancers such as liver, lungs, brain, and so on, to prolong the survival rate and revamp the quality of life. Indeed, an in-depth knowledge of curcumin-free compounds through these clinical trials help to understand that non-curcuminoids are powerful compounds that have the capability of treating various diseases and can even be more beneficial if impregnated in various formulations revolving liposomes, emulsions, solid lipid nanoparticles, and so on. Large-scale trials should be conducted to prove the potency of non-curcuminoid drug delivery formulations, which might be beneficial to assimilate an approval for human use [120].

## 6. Biological Activities of Non-Curcuminoids in Different Drug Delivery Formulations

**Figure 4:**
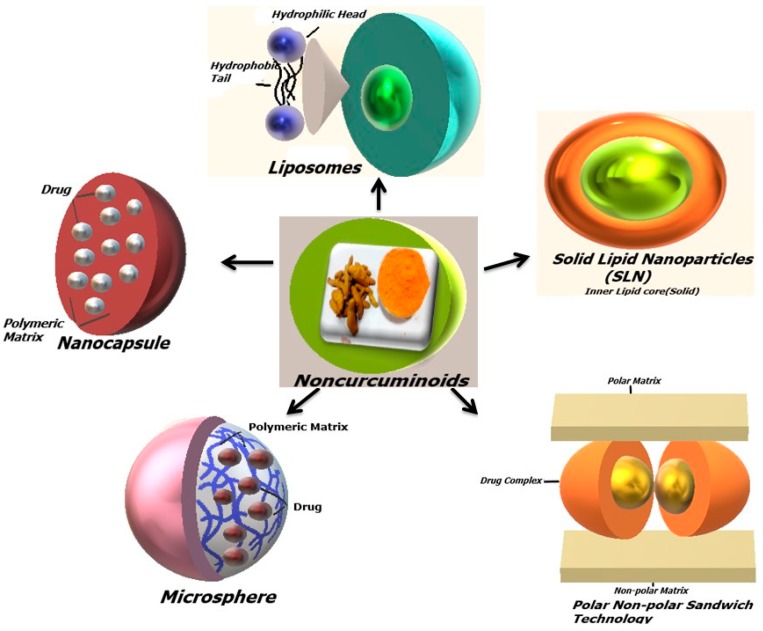
Various encapsulation techniques for non-curcuminoids in different drug delivery formulations.

Studies have proved that non-curcuminoids have a frontline implicit to subdue cancer, but low bioavailability and poor aqueous solubility have always remained as a major concern. Thus, researchers from the past few decades are continuously functional to surmount these disadvantages, by incorporating them in different delivery system to facilitate target delivery (Figure 4) (Table 3).

### 6.1. Cocrystals

Cocrystals are homogenous crystalline formulations comprising of two or more compounds in which one of the active moieties are in the form of definite stoichiometric crystal structure bonded by noncovalent and nonionic bonds comprising of hydrogen bonds. These cocrystals prove to be advantageous in altering the physicochemical properties increasing the hygroscopicity, solubility, dissolution, and stability. This formulation also facilitates combination therapy such as drug-herb, drug-drug, and herb-herb, which help to improve patient compliance and diminish adverse effects. Many attempts have come forth for successful cocrystalization of turmeric constituent particularly curcumin. Suitable coformers of benezenetriols and benezenediols such as resorcinol, catechol, hydroxyquinol, hydroquinone, phloroglucinol, and pyrogallol were employed, among which pyrogallol and resorcinol formed successful hydrogen bond (O-H---O) in between hydroxyl phenol group and keto functional groups of curcumin enriching the physicochemical properties of curcumin. Various methods have also been developed up until now, mainly, rapid solvent evaporation to form pure phase crystals with curcumin. In addition, profound improvement of curcumin was demonstrated in contrast with individual coformers in terms of its hygroscopicity, stability and dissolution [120]. It is believed that the linker (β-diketone) in the seven-carbon chain of curcumin is the key factor responsible for its low solubility and reaction with this keto-enol form can be altered by tailoring intermolecular hydrogen bonds with coformers through cocrystalization Hence, these cocrystals provide promising platform for future development of curcuminoids, as well as non-curcuminoids, particularly compounds which are structurally similar to curcumin, like cyclocurcumin and calebin A, to be tailored as solid dosage form with immense solubility and stability [120,121].

### 6.2. Liposomes

Liposomes are spherical bi-layered vesicles having phosphate head of phospholipids, which are hydrophilic and fatty acid tails (lipids), which are hydrophobic. There were many liposomal non-curcuminoids formulations studied recently with β-elemene and furanodiene. Chen et al. explained various formulations including liposomes of β-elemene and zedoary turmeric oil. Their formulation helped prolong the circulation of turmeric oil liposomes, reduce the irritation and improved the efficacy of β-elemene by encapsulating them in PVP-coated liposomes. Moreover, in vivo studies revealed that liposomal β-elemene were distributed in spleen, kidney, and liver in rats after intravenous administration. Another study summarized the recent advances in the liposomal delivery system of β-elemene exhibiting anticancer activity, mainly on lung cancer because of the various benefits such as availability, angiogenesis inhibition, induction of apoptosis, and enhancement of radiosensitivity. It also exhibited chemotherapeutic effects when combined with other anticancer agents and exhibited minimal side effects, such as damage of kidney and liver function. System biology, network pharmacology and clinical trials provided adequate evidence of β-elemene as a potent anti-lung cancer agent. This also proved that the advancement in molecular pharmaceutical technologies provided a broad platform for making it suitable for liposomal-based delivery [122]. Furanodiene hold diverse pharmacological activities ranging from high lipophilicity to poor stability. Comparative research by loading furanodiene with biodegradable polylactide-co-glycolide (PLGA) and pegylated PLGA nanoparticles showed improvement in the stability, hydrophilicity and bioavailability of furanodiene, thus facilitating the permeation of furanodiene across Caco-2 cell monolayers [123].

Furthermore, FN-loaded FA-PEG2000-DSPE-modified nanostructured lipid carriers (FA-FN-NLCs) were developed by using emulsification-ultrasonic and low temperature solidification method, optimized with central composition design, to increase the solubility and bioavailability of FN. The advantages being, increased circulation time in blood and improved targeting ability. Hence, this formulation emerged as an optimistic drug delivery system for FN in the treatment of cancer exhibiting high encapsulation efficiency and loading capacity. Even, through NiR fluorescence imaging study, it was seen that FA-FN-NLCs especially accumulated in tumor site, by receptor-mediated endocytosis. In vitro drug release exhibited a biphasic release pattern with an initial sudden drug release and followed by a prolonged drug release. In vivo study compared with FN solution (FN-SOL) and FN loaded traditional NLCs (FN-NLCs) and showed that FA-FN-NLCs had a longer blood circulating time (t_1/2_) and higher AUC [124]. Recently, Pan et al. compared calebin A and calebin A encapsulated liposomes and concluded that liposomes of calebin A increased the bioactivity of calebin A by enhancing its anticancer activity in colon cancer. This study explained that, besides enhanced solubility, liposomal calebin A has the potency to change absorption pathway and signaling processes in an increased manner, establishing it as a promising agent in inhibiting colon cancer (SW480) cells [125]. Therefore, these studies explain the significant increase in the anticancer activities of non-curcuminoids through liposomal formulation.

### 6.3. Solid Lipid Nanoparticles

Solid lipids nanoparticles are solid colloidal particles in which the active agents or drugs are entrapped and dissolved. Researches showed that β-elemene, prepared in solid lipid nanoparticulate (SLN) system with various methods such as film ultrasonic wave, sonication and extrusion attained various benefits: achieving sustained release pattern by improving the protection of labile compounds from chemical degradation, increasing the availability and efficiency of the drug. Another research showed a comparison of normal SLN and folate receptor-targeted SLN encapsulated turmeric oil and β-elemene as well as observed that the later circulates for a longer period in blood as well as in many tissues to reach higher level of concentration [122]. Hence, non-curcuminoid encapsulated solid lipid nanoparticles can be beneficial to enhance their anticancer activities.

### 6.4. Nanocapsules

Nanocapsules are nanoscale shell made from non-toxic polymeric membrane, which encapsulates an inner liquid core. Natrajan et al. developed chitosan-alginate nanocarrier for the encapsulation of ar-turmerone, which resulted in a clear supernatant and through hemolysis assay, confirmed that they were hemocompatible. The carrier showed high cell viability percentage, which insinuate it as nontoxic and with nanoscale shell. Moreover, turmeric oil loaded nanocapsules were found to have antiproliferative activity on A549 cell lines by using MTT assay [126]. Hence, this suggests that non-curcuminoid loaded nanocapsules may also accelerate their anticancer activities.

### 6.5. Microcapsule/Microsphere

Microcapsules are formed by incorporation of active agent in a polymeric matrix. A research showed that both β-elemene and zedoary turmeric oil encapsulated in microcapsules have marked improvement in their therapeutic efficacy. In addition, microspheres are free flowing powders consisting of synthetic polymers or proteins. These oil microspheres have great anticancer potential in transplanted hepatoma, which is used in the treatment of hepatic artery infusion, to relieve the abnormality of blood cell count caused by mitomycin [122]. Hence, these nanoparticles are promising in hepatoma cancer to enhance the bioavailability of non-curcuminoids and improve their anticancer activities.

### 6.6. Microemulsion and Nanoemulsion

Microemulsions are clear and thermodynamically stable; they are formed by isotropic liquid mixtures of oil, water and surfactant, where oil phase contains different olefins and hydrocarbons whereas aqueous phase contains salt or other constituents. Ju et al. proved that elemene microemulsions are beneficial for the treatment of malignant neoplasms, such as gynecological, breast, skin, lung, and gastrointestinal cancers and, hence, exhibit sustained release in vivo with improved bioavailability, stability, high entrapment efficiency and good clarity. In addition, it was found that submicron emulsion possessed high turmeric oil loading capacity. This can stay stable and sterilized at room temperature, as well as under high temperature, pressure for six months [122]. Gao et al. through in vivo studies suggested that β-elemene nanoemulsion inhibited the evolution of hepatoma 3B cells more efficiently in contrast to β-elemene water solution in a concentration-dependent manner [127]. Thus, micro and nano emulsions improve non-curcuminoids bioavailability and solubility to ameliorate their anticancer activities.

### 6.7. Complete Natural Turmeric Matrix Formulation

Our research group developed a more bioavailable form of curcuminoids—Cureit—formed by recreating a complete natural turmeric matrix (CNTM) using a novel PNS technology. This CNTM contains not only curcuminoids; but also, non-curcuminoids in the form of turmeric water extract and turmeric essential oil [118,128]. The presence of non-curcuminoids in Cureit intensifies the physical stability and facilitates sustained drug delivery along with the protection of drug molecules from degradation [129]. The CNTM significantly improves the symptoms and biomarkers of rheumatoid arthritis patients due to the anti-inflammatory properties of CNTM containing curcuminoids and non-curcuminoids [119]. This CNTM can also use as an anti-ageing, antioxidant and anticancer agent [130,131,132]. Hence, this novel nano drug delivery system promises to effectively encapsulate non-curcuminoids with curcumin without any drug interactions. Indeed, it is a successful system for the synergistic combination of non-curcuminoids with curcumin, which ameliorate the bioavailability and enhance the anticancer activities without rendering any adverse effects.

## 7. Conclusions

Non-curcuminoids are basically all other biologically active compounds of turmeric excluding curcuminoids. The latter has been well established in the research field for promoting green drug discovery. However, this review attempts to resolute the foundation for non-curcuminoids by highlighting a literature-based summary on their dynamic anticancer potential. The discovery, chemical composition including the structure and physicochemical properties of these bioactive compounds provide a useful scaffold of knowledge about each component. In addition, certain non-curcuminoids have shown similarity in their chemical structures as curcuminoids for example cyclocurcumin and calebin A. Some major non-curcuminoids such as bisacurone, elemene, turmerones, furanodiene, germacrone, curcumol, calebin A and curdione are considered in this review, which help apprehend that, the anticancer activities of these compounds that can be comparable to curcuminoids. Indeed, the alteration in various signaling pathways such as NF-ĸB, TNF and inhibition of enzymes such as COX-2, protein kinase, cytokines and MMPs transcription factors as well as blocking cancer cell proliferation at different phases of cell cycle such as G1, G2, M, S to induce apoptosis, are comparable to the cancer fighting mechanism of curcuminoids. Further, modulation of p-glycoprotein in non-curcuminoids could increase the uptake by the cells; help them to act synergistically with curcumin. Moreover, the molecular docking studies of elemene, germacrone, cyclocurcumin have been discussed which gives a thorough knowledge of the most favorable binding sites of these compounds, which reveal that these compounds hold a promising place as an anticancer agent to fight against various cancers. These similarities in structure and mechanism of action prove that non-curcuminoids are equipotent and highly effective as curcuminoids and can be used in combination or alone with the latter. Additionally, both are extracted from turmeric with certain variation in the end process and hence, these compounds provide an added perquisite to be established in the medicinal world.

Moreover, various preclinical and clinical studies of these compounds were conducted to have an extensive understanding to recondite the mechanism of action and anticancer activities on in vivo rat and human models. However, these compounds possess certain physicochemical issues particularly issues pertaining to its low solubility and bioavailability, therefore, attempts to revamp the anticancer activities by numerous encapsulation techniques help conceal the debits of these curcumin-free compounds. Proper selection of delivery systems for non-curcuminoids are promising, especially with PNS technology, which has successfully identified these compounds, along with curcumin components without any distortion of shape or size. In fact, this technology is a feather in the cap of these versatile non-curcuminoids, which prove promising benefits when used in combination with a more bioavailable form of curcumin. Clinical studies were also conducted on these formulations based non-curcuminoids which consolidate promising results that make them strong contender in the medicinal market to fight numerous diseases especially cancer. However, more studies on the exclusive therapeutic benefits of these components through more clinical trials on varied catalogs such as by considering larger group of cancer patients, extending treatment regimen duration and monitoring safe effective dose can promulgate its universal acceptance. This review tries to unveil the promising effects of non-curcuminoids by giving an insight on researches conducted up until now. Nevertheless, more studies including the bioavailability, drug interactions, effect on multi drug resistance and chemopreventive activities when used alone or in combination with other cancer treatment drug can provide them as an epitome of economical cure and relief in the discipline of medicine. These benefits acknowledge a wide scope for various cancers, late or early stage cancers and recurrent cancers, which would help in the decline of relapses and death rate. To summarize, this review is an integrative approach to promote these natural compounds as an affluent and benevolent source for future studies.

## Figures and Tables

**Table 1:** Chemical structures and physicochemical properties of selected non-curcuminoids.

Name	IUPAC Name	Structure	Molecular Weight (g/mol)	Molecular Formula	Physical and Chemical Properties
Ar-turmerone	6S-2-methyl-6-(4-methylphenyl) hept-2-en-4-one	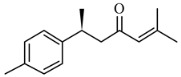	216.324	C_15_H_20_O	Appearance: Yellowish oil Boiling point: 326 °C Melting point: 122 °C Density: 0.945 g/cm^3^ Solubility: Hexane, petroleum ether, ethanol
β-Elemene	(1S,2S,4R)-1-ethenyl-1-methyl-2,4-bis(prop-1-en-2-yl) cyclohexane	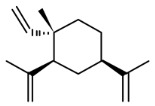	204.357	C_15_H_24_	Appearance: Colorless to yellow clear liquid Boiling point: 252 °C Melting point: 98 °C Density: 0.862 g/cm^3^ Solubility: Alcohol
γ- Elemene	(1S,2S)-1-ethenyl-1-methyl-2-propan-2-ylidene-2-prop-1-en-2-yl) cyclohexane	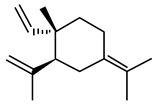	204.357	C_15_H_24_	Appearance: Clear and colorless liquid Boiling point: 258.2 ± 40 °C at 760 mmHg Melting point: 101.3 ± 22.2 °C Density: 0.9 ± 0.1 g/cm^3^ Solubility: Alcohol
Furanodiene	(5E,9E)-3,6,10-trimethyl-4,7,8,11-tetrahydrocyclodeca(b) furan	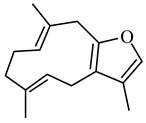	216.324	C_15_H_20_O	Appearance: White powder Boiling point: 309.6 ± 11 °C at 760 mmHg Melting point: 137.1 ± 6.1 °C Density:0.9 ± 0.1 g/cm^3^ Solubility: Partially soluble in water
Calebin A	(E-4-(4-hydroxy-3-methoxyphenyl)-2-oxobut-3-enyl)E-3-(4-hydroxy-3-methoxyphenyl)prop-2-enoate)	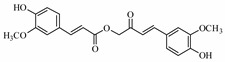	384.384	C_21_H_20_O_7_	Appearance: White powder Boiling point: 592.1 ± 50.0 °C at 760 mmHg Melting point: 207.9 ± 23.6 °C Density:1.3 ± 0.1 g/cm^3^ Solubility: Sparingly soluble in water
Germacrone	(3E,7E)-3,7-dimethyl-10-propan-2-ylidenecyclodeca-3,7-dien-1-one	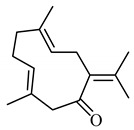	218.34	C_15_H_22_O	Appearance: White crystalline powder Boiling point:666.52 °C Melting point:347.53 °C Density:0.9 ± 0.1 g/cm^3^ Solubility: Methanol
Cyclocurcumin	(E)-2-(4-hydroxy-3-methoxyphenyl-6-(4-hydrox-3-methoxystyryl)-2H-pyra-4(3H)-one	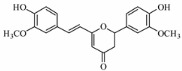	368.38	C_21_H_20_O_6_	Appearance: Yellow powder Boiling point:571.9 ± 50.0 °C at 760 mmHg Melting point: 202.6 ± 23.6 °C Density:1.4 ± 0.1 g/cm^3^ Solubility: Ethanol and concentrated acetic acid
Bisacurone	(6S)-6-((1R,4S,5S)-4,5-dihydroxy-4-methylcyclohex-2-en-1-yl)	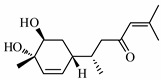	254.354	C_15_H_24_O_3_	Appearance: Bright orange powder Boiling point: 377.7 ± 42.0 °C at 760 mmHg Melting point: 196.4 ± 24.4 °C Density: 1.1 ± 0.1 g/cm^3^ Solubility: Acetone and ethanol
Curcumol	(1S,2S,5S,9S)-9-isopropyl-2-methyl-6-methylene-11-oxatricyclo(6.2.1.0^1,5^)undecan-8-ol	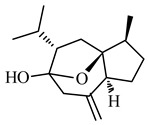	236.355	C_15_H_24_O_2_	Appearance: White crystals Boiling point:334.5 °C Melting point:134.69 C Density:1.06 g/cm^3^ Solubility: DMSO in ethanol

**Table 2:** Various anticancer activities of non-curcuminoids.

Non-Curcuminoids	Various Anticancer Studies	References
Turmerone	Unlike curcuminoids, turmerone brought apoptosis due to the distortion caused to the mitochondria in the intrinsic pathway with activation in procaspase-3 cleavage	[33]
Ar-turmerone	Ar-turmerone brought apoptosis on various cell lines such as K562, RBL-2H3, L1210, and U937 in time and dose-dependent manner.	[34]
	Ar-turmerone facilitated curcumin transport through heterogeneous human epithelial colorectal adenocarcinoma (Caco 2) cells and inhibited the efflux of rhodamine-123 and permeability glycoprotein multidrug resistant gene messenger ribonucleic acid (Pgp (MDRI gene) mRna) expression levels.	[35]
	Ar-turmerone suppressed 12-O-tetradecanoylphorbol-13-acetate (TPA)- induced up-regulation of matrix metalloproteinase -9 (MMP-9) and cyclooxygenase-2 (COX-2) expression by blocking nuclear factor kappa light chain enhancer of activated B cells (NF-kB), Phosphoinositide 3-kinase/ protein kinase B (PI3K/Akt) and extracellular regulated kinase (ERK)1/2 signaling in human breast cancer cells and inhibited TPA induced invasion, migration, and colony formation in human breast cancer cells.	[36]
	Ar-turmerone was effective on murine dendritic cells (DCs).	[37]
	Ar-turmerone showed inhibitory action against hepatocellular carcinoma cells by apoptosis through intracellular reactive oxygen species (ROS) generation-mediated activation of ERK and c Jun N terminal kinase (JNK) kinases.	[38]
	Ar-turmerone had cytotoxic effects on Lymphocytic leukemia (L-1210) and myeloid cell line (HL-60) cells with an inhibition rate of 11–12% and apoptosis index 5–6.	[39]
	Ar-turmerones enhanced the activities of curcumin in human colon carcinoma (HCT-116), colorectal adenocarcinoma (HT-29) and human umbilical vein endothelial cells (HUVEC) cell lines.	[40]
	Ar-turmerone, when motif-fused with spiropyrollidone oxindoles increased the potency in lung cancer and leukemia cells.	[41]
	Ar-turmerone and its analogs exhibited cytotoxic activities against L1210 cell.	[42]
	Ar-turmerone exhibited apoptosis through changes in morphological characters.	[43]
	Ar-turmerone exhibited apoptosis on human myeloid leukemia HL-60 cells.	[44]
Elemene	Elemene can inhibit the tumor growth of various cells, such as ovarian, laryngeal, non-small cell lung, prostrate, melanoma, leukemia, breast, brain, hepatoma, colorectal adenocarcinoma, glioblastoma, and human cervix epithelioid carcinoma cells	[45]
	Elemene brought apoptosis in HL-60 cell by causing cell cycle arrest between phase transitions from S to G2M phase	[46]
	Elemene can pass through the blood brain barrier due to its small size and lipophilic nature which is helpful in brain carcinomas	[47]
β-elemene	The mechanism of action of β-elemene in non-small-cell lung cancer cell (NSCLC) death may be through a mitochondrial release of the cytochrome c-mediated apoptotic pathway.	[48]
	β-elemene sensitizes the human NSCLC cell lines H460 and A549 to cisplatin via mitochondria mediated intrinsic apoptosis pathway involving B cell lymphoma-2 (Bcl-2) family proteins and IAPs (inhibitor of apoptosis proteins) in a time and dose-dependent manner.	[49]
	β-elemene exhibited antitumor effect on NSCLC A549 cells and inhibited the activity of the PI3K/Akt/mammalian target of rapamycin (mTOR)/Ribosomal protein S6 kinase beta-1(p70S6K1) signaling pathway, resulting in apoptosis as well as protective autophagy.	[50]
	β-elemene inhibited ERK and Akt activation and upregulation of Casitas B–lineage lymphoma (c-Cb1 and Cb1-b) expression to initiate apoptosis in lung cancer cells.	[51]
	β-elemene has potential to inhibit human lung cancer NSCLC cell growth via ERK1/2- and AMP-activated protein kinase (AMPKa)-mediated inhibition of transcription factor Sp1, followed by reduction in DNA (cytosine-5)-methyltransferase 1(DNMT1) protein expression.	[52]
	β-elemene in combination with etoposide is beneficial for lung cancer cells.	[53]
	β-elemene is a promising therapeutic role in human SGC7901 and MKN45 gastric cancer cells.	[54]
	β-elemene and its relation between the expression level of tyrosine-protein kinase Met (c-Met) promoted its anticancer mechanism.	[55]
	β-elemene can inhibit the proliferation of RCC 786- 0 cells by the inhibition of mitogen activated protein kinase (MAPK)/ERK and PI3K/Akt/mTOR signaling pathway, thus inducing apoptosis and protective autophagy.	[56]
	β-elemene when treated with taxane over ovarian cell lines A2780/CP70 and its parental cell line A2780 showed promising results.	[57]
	β-elemene can reverse tumor multi drug resistance (MDR) and enhanced the doxorubicin activity.	[58]
	β-elemene showed antiproliferative activity on glioblastoma cells which was supported by phosphorylation of p38 MAPK, cell-cycle arrest in the G0/G1 phase.	[59]
	β-elemene inhibited the proliferation and survival of the cell lines of glioblastoma multiforme (GBM) when combined with radiotherapy or temozolomide (TMZ) via inhibition of DNA damage repair.	[60]
	β-elemene arrested glioblastoma cells (C6 and U87) in G0/G1 phase of the cell cycle, as well as brought about inhibition in cell proliferation by regulating the glia mutation factor β/mitogen activated protein kinase 3/6/p38 and extracellular signal-regulated kinase 1/2/B cell lymphoma 2/surviving pathways.	[61]
	β-elemene enhances susceptibility to cisplatin resistant ovarian cancer cell.	[12]
	β-elemene exhibited antiproliferative activities, promoted apoptosis, impaired invasiveness in glioblastoma cells, suppressed the growth of animal xenografts.	[62]
	β-elemene and its derivatives containing a piperazine, a morpholine, a tetrahydropyrrole, a thiophenylethylamine, or a cyclohexamine group exhibited proliferative activity in human cervix epitheloid carcinoma HeLa, gastric carcinoma SGC-7901, and leukemia K562 cells.	[63]
	β elemene monosubstituted amine, ether and rhenium coordinated complex structure was synthesized and characterized. The in vitro anti-proliferative activity of β-elemene monosubstituted amine and Re (CO)_3_-β-elemene derivatives in human cervix epitheloid carcinoma HeLa cells were promising.	[64]
	β-elemene-enhanced inhibitory effect of cisplatin on lung carcinoma cell proliferation, regulated by a check point kinase (CHK) 2-mediated cell division cycle CDC25C/CDC2/cyclin B1 signaling pathway which leads to the blockade of cell cycle progression at G2/M.	[65]
	Arsenic trioxide (ATO) combined with the derivative of β-elemene, *N*-(β-elemene-13-yl) tryptophan methyl ester (ETME) synergistically enhanced the antiproliferative activity and apoptosis in hepatocellular carcinoma (HCC) cells.	[66]
	Novel furoxan-based NO- donating β-elemene hybrids are promising anti-cancer agents.	[67]
	Comparison of cytotoxic efficacy of β-elemene and its synthetic analogs [(R or S)–2–((1R,3S,4S)–3–isopropenyl–4–methyl–4–vinylcyclohexyl)–propane–1,2–diol] (Lr-1), [(S)–2–((1R,3S,4S)–3–isopropenyl–4–methyl–4–vinyl–cyclohexyl)–propane–1,2–diol and (R)–2–((1R,3S,4S)–3–isopropenyl–4–methyl–4–vinylcyclohexyl)–propane–1,2–diol] Lr-2) in the brain tumor cell lines A172, CCF-STTG1, and U-87MG proved beneficial.	[68]
	β-elemene was reviewed for its anticancer effects on different cancer cells and apoptosis and cell cycle arrest were found to be the major cause behind its anticancer activities β-elemene is influential in altering the functionality and quantity of ABC transporters thereby efficient in doxorubicin resistant breast cancer cells.	[69,70]
	The possible mode of anticancer activity of β-elemene in altering MDR through the inhibition of ABCB1 transporter efflux activity was determined through molecular docking study	[71]
	Docking studies proved that sesquiterpenes have potential biological activities	[72]
δ-elemene	Increment of p38 MAPK and inducible nitric oxide synthase levels were seen when δ -elemene was treated in NCL-H292 lung cancer cells through activation of the caspase signaling pathway.	[73]
Furanodiene	Furanodiene were useful in the treatment of liver diseases.	[74]
	Furanodiene was influential on human leukemia HL60 cells, evaluated by DNA fragmentation.	[75]
	Furanodiene is an effective agent against uterine cervix cancer, and has a protective effect on the immune function.	[76]
	Furanodiene induced cell death in dose-dependent manner when analyzed by (3-(4,5-dimethylthiazol-2-yl)-2,5-diphenyltetrazolium bromide) tetrazolium reduction assay MTT assay, thus effective against uterine cervical cancer growth.	[77]
	Bioavailability of furanodiene in rat’s plasma as analyzed with liquid chromatography/tandem mass spectrometry was about 49.0%.	[78]
	Furanodiene suppresses breast cancer cell growth both in vitro and in vivo in a dose-dependent manner inducing cell cycle arrest at the G0/G1 phase.	[79]
	Furanodiene increases the inhibition activities and apoptosis nature of tamoxifen, thus facilitating the treatment of breast cancer. It was beneficial against proliferation carried out by vascular endothelial growth factor (VEGF).	[80]
	Furanodiene showed antiproliferative activity on A549, NIH-H1299, and 95-D lung cancer cells and was useful in combination therapy with paclitaxel.	[81]
	Growth inhibitory and pro-apoptosis activity of furanodiene was enhanced and affected Tamoxifen (TAM) by inducing cell cycle arrest and cell apoptosis.	[82]
	Furanodiene suppresses proliferation and increase the lactate dehydrogenase (LDH) release in a dose-dependent manner by cell cycle arrest at G0/G1 phase and apoptosis in breast cells.	[83]
	Doxorubicin-resistant MCF-7 (MCF-7/DOXR) breast cancer cells, when treated with furanodiene showed an alteration in mitochondrial function as well as adenosine triphosphate (ATP) levels were reduced causing apoptotic cell death.	[84]
	The combinational treatment of furanodiene with doxorubicin in breast cancer cells were found promising.	[85]
Furanodienone	Furanodienone inhibited cell cycle proliferation and induced apoptosis in dose-dependent manner by inhibiting estrogen receptor alpha signaling alpha and mRNA expression levels without effecting estrogen receptor (ER) beta.	[86]
	Furanodienone also triggered apoptosis on human breast cancer cells through epithelial growth factor pathways.	[87]
	Furanodienone induced apoptosis through ROS on colorectal carcinoma cells.	[88]
Curcumol	Curcumol effects cell proliferation by RNA synthesis in a concentration-dependent manner in lung cancer.	[89]
	Curcumol bring apoptosis in nasopharyngeal carcinoma CNE-2 cells by down regulation of NF-kb.	[90]
	Curcumol induced apoptosis by suppression of PI3K/Nf-kB pathway on hepatic stellate cells.	[91]
	Curcumol exhibited promising anticancer effects when treated on colorectal cancer cells (LOVO).	[92]
Cyclocurcumin	Cyclocurcumin inhibited the proliferation of breast cancer cells.	[12]
	When combined with curcumin, cyclocurcumin showed nematocidal behavior.	[93]
	Trans-to-cis isomerization of cyclocurcumin proved beneficial.	[94]
	Through docking study, an insight on the efficiency of cyclocurcumin as therapeutically potential compound for treating various cancers such as ovarian, brain, lymphomas (Hodgkin and non-Hodgkin), lungs, and adrenocortical cancers was provided	[95]
Calebin A	Calebin A inhibited growth and induced apoptosis in drug-resistant human gastric cancer cells reduction in S phase and G2/M phase arrest.	[96]
	Calebin A inhibited growth in drug-resistant human colon cancer cells by decreasing the expression of cell cycle regulatory protein and increasing the ROS levels, inducing apoptosis.	[97]
	Calebin A has wide scope in multiple myeloma and breast cancer cells by suppression of osteoclastogenesis.	[98]
Germacrone	Germacrone inhibited human breast cancer cells by cell cycle arrest and apoptosis with increase in the LDH release and inducing mitochondrial membrane potential depolarization.	[99]
	Germacrone inhibits human hepatoma cell lines by protein expression of cyclin B1 decrease and activation of cyclin-dependent kinases inhibitors (CDK)1.	[100]
	Germacrone shared relation between the JANUS-activated kinases (JAK2)/ Signal transducer and activator of transcription (STAT) 3 signaling pathway and it induced apoptosis in HepG2 cells.	[101]
	The combination of germacrone with ADR enhanced the apoptotic effect and resulted in the reduction of anti-apoptotic protein expression levels (Bcl-2) and enhancement of pro-apoptotic protein expression levels (p53 and Bcl 2 associated X protein (Bax) in MCF-7/ADR cells.	[102]
	Germacrone is beneficial in combination therapy with other drugs as it potentiates the anti- tumor activity of methotrexate and 5-fluorouracil on ER α-positive breast cancer cells.	[103]
	The derivatives of germacrone had inhibitory effects on Bel-7402, HepG2, A549, and HeLa cells.	[104]
	Germacrone possess enormous biological activities as proved by molecular docking study	[105]
Bisacurone	Bisacurone inhibits adhesion of cancer cells to endothelial cells through down regulation of vascular cell adhesion molecule (Vcam)1 expression.	[106]
Curdione	Curdione inhibited production of prostaglandin (PG), E2 in lipopolysaccharide (LPS)-stimulated mouse machrophages RAW 264.7 through suppression of enzyme COX-2 mRNA expression in a dose-dependent manner.	[107]
	The three main sesquiterpenes (germacrone, curdione, furanodiene) had proliferative activity on MDA-MB-231 and MCF-7 breast cancer cells, alone or in combination with a fixed-dose-combination.	[108]
	Curdione inhibited the proliferation of breast cancer cells in xenograft nude mouse in a dose-dependent manner.	[109]
	Additionally, the pharmacokinetic studies of curdione proved promising.	[110]

Abbreviations: ADR, Adriamycin; AMPKa, AMP-activated protein kinase; ATO, arsenic trioxide; ATP, adenosine triphosphate; Bax, Bcl 2 associated X protein; Bcl, B cell lymphoma; CB1, Casitas B–lineage lymphoma; Caco-2, heterogeneous human epithelial colorectal adenocarcinoma cells; CDC, cell division cycle; CHK2, checkpoint kinase; c-Met, tyrosine-protein kinase Met; COX-2, cyclooxygenase-2; DC, dendritic cells; DNMT1, DNA (cytosine-5)-methyltransferase 1; DOXR, doxorubicin; ERK1/2, extracellular regulated kinase; ER, estrogen receptor; ETME, N-(β-elemene-13-yl) tryptophan methyl ester; HCC, hepatocellular carcinoma; GBM, glioblastoma multiforme; HCC, hepatocellular carcinoma; HCT, human colon carcinoma; HL-60, myeloid cell line; HT, colorectal adenocarcinoma; HUVEC, human umbilical vein endothelial cells; JAK, Janus activated kinases; JNK, c Jun N terminal kinase; L1210, lymphocytic leukemia; LDH, lactate dehydrogenase; LPS, lipopolysaccharide; LOVO, colorectal cancer cell; Lr-1, [(R or S)–2–((1R,3S,4S)–3–isopropenyl–4–methyl–4–vinylcyclohexyl)–propane–1,2–diol]; Lr-2- [(S)–2–((1R,3S,4S)–3–isopropenyl–4–methyl–4–vinyl–cyclohexyl)–propane–1,2–diol and (R)–2–((1R,3S,4S)–3–isopropenyl–4–methyl–4–vinylcyclohexyl)–propane–1,2–diol]; MAPK, mitogen activated protein kinase; MDR, multi drug resistance; MMP-9, matrix metalloproteinase-9; mTOR, mammalian target of rapamycin; MTT assay, (3-(4,5-dimethylthiazol-2-yl)-2,5-diphenyltetrazolium bromide) tetrazolium reduction assay; NCL-H292, mucoepidermoid lung cancer cell; NF-kB, nuclear factor kappa light chain enhancer of activated B cells; NSCLC, non-small cell lung cancer cell; P-gp (MDRI gene) mRNA, permeability glycoprotein multidrug resistant gene messenger ribonucleic acid; PG, prostaglandin; PI3K/Akt, phosphoinositide 3-kinase/ protein kinase B; P70S6K1, ribosomal protein S6 kinase beta-1; ROS, reactive oxygen species; STAT, signal transducer; TAM, tamoxifen; TMZ, Temozolomide; TPA, 12-O-tetradecanoylphorbol -13-acetate; VEGF, vascular endothelial growth factor; Vcam, vascular cell adhesion molecule.

**Table 3:** Various formulations of non-curcuminoids and their biological activities.

Formulation	Method	Active Ingredient	Major Activities	References
Co-crystals	Rapid solvent evaporation	Curcuminoids	Enhance hygroscopicity, stability, dissolution	[120]
	Rapid solvent evaporation	Turmeric compounds	Enhance solubility	[121]
Liposomal products	Freeze dry method	Turmeric Oil	Anticancer activity	[122]
	Thin film hydration followed by reverse phase evaporation with high-pressure extrusion	β-elemene		
Pegylated liposomes	Emulsification by ultrasonication and solidification through low temperature	Furonodiene	Anticancer activity	[123,124]
	Thin film sonication method	Calebin A	Anticancer activity	[125]
Solid lipid nanoparticles	Membrane extrusion and Supersonic film ultrasonic wave dissolving technique	β-elemene	Enhance the bioavailability of active molecules	[122]
Nanocapsules	Encapsulation	Ar-turmerone	Antiproliferative activity	[126]
Microcapsule/Microsphere	Emulsification internal gelatification technology	Zedoary turmeric oil and β-elemene	Antitumor activity	[122]
Microemulsion	High pressure emulsification with phase inversion ultrasound technology	Zedoary turmeric oil and β-elemene	Anticancer agent for lung, breast, gastrointestinal, skin and gynecological cancer cells	[122]
	Phase inversion method	β-elemene	Anticancer agent for hepatoma 3B cells cancer cells	[127]
PNS Cureit	Polar-nonpolar-sandwich (PNS) technology	Complete natural turmeric matrix	Anti-aging, Anti-rheumatoid activity	[118,119,128,129,130,131,132]

## Abbreviations

ACLY	ATP citrate lyase
ACP	Acidic phosphatase
ADR	Adriamycin
AICAR	(5-Aminoimidazole-4-carboxamide-1-b-4-ribofuranoside
Akt	Protein kinase B
AMPK	Adenosine monophosphate-activated protein kinase
AMPKa	AMP-activated protein kinase
Ar-turmerone	Aromatic turmerone
ATM	Ataxia telangiectasia mutated
ATO	Arsenic trioxide
Bax	Bcl 2-associated X protein
Bcl	B cell lymphoma 2
BCRP	Breast cancer resistance protein
Bid	BH3-interacting domain death agonist
BIP	Binding immunoglobulin protein
Cdc	Cell division cycle
CDKI	Cyclin-dependent kinases inhibitors
cFLIP	Cellular FLICE-like inhibitory protein
CHK2	Checkpoint kinase 2
cMet	Tyrosine-protein kinase Met
CNTM	Complete natural turmeric matrix
COX-2	Cyclooxygenase -2
CREB	Camp-response element
CTR	Calcitonin receptor
DC	Dendritic cells
DNA	Deoxyribonucleic acid
DNMT1	DNA (cytosine-5)-methyltransferase 1
DOXR	Doxorubicin
DR4	Death receptor 4
ED50	Effective dose—50%
EGFR	Epithelial growth factor
ELISA	Enzyme-linked immunosorbent assay
EMT	Epithelial mesenchymal transition
ER	Endoplasmic reticulum
ERK	Extracellular regulated kinase
ER	α-Estrogen response
ETME	N-(β-Elemene-13-yl) tryptophan methyl ester
FACS	Fluorescence activated cell sorting
FAS	First apoptosis signal
FITC	Fluorescein isothiocyanate
FN	Furanodiene
GBM	Glioblastoma multiform
GFAP	Glial fibrillary acidic protein
GSCs	Glioblastoma stem cells
GSK	3-Glycogen synthase kinase
HCC	Hepatocellular carcinoma
HER	Human epithelial growth receptor
HIF-1	Hypoxia-inducible factors
HSC	Hepatic stellate cells
HUVEC	Human umbilical vein endothelial cells
IAPs	Inhibitor of apoptosis protein
IARC	The international agency for research on cancer
IC50	Half-maximal inhibitory concentration
IGF-1R	Insulin-like factor-1 receptor
IL2	Interleukin 2
JAK	JANUS-activated kinases
JNK	c Jun N terminal kinase
LDH	Lactate dehydrogenase
LPS	Lipopolysaccharide
MAPK	Mitogen activated protein kinase
MDR	Multi-resistance protein gene
MKK	Mitogen-activated protein kinase kinase
MMP-9	Matrix metalloproteinase -9
MMP	Mitochondrial membrane potential
mRNA	Messenger ribonucleic acid
Myc	Myelocytomatosis
NAC	N-acetyl cysteine
NF-κB	Nuclear factor kappa light chain enhancer of activated B cells
NFATc-1	Nuclear factor of activated T-cells, cytoplasmic 1
NLC	Nanostructured lipid carriers
NSCLC	Non-small cell lung cancer cell
N-WASP	Neural Wiskott-Aldrich syndrome protein
PARP	Poly (ADP-ribose) polymerase; PARP
PCNA	Protein cell nuclear antigen
P-gp	Permeability glycoprotein
Phosphor-cdc2	Phosphorylated cell division cycle 2
PKC	Protein kinase C
PLGA	Poly (lactic-co-glycolic acid)
PUMA	p 53 upregulated modulator of apoptosis
RANKL	Receptor activator of nuclear factor kappa-Β ligand
ROS	Reactive oxygen species
RT-PCR	Reverse transcription polymerase chain reaction
SHH	Sonic hedgehog
SLN	Solid lipid nanoparticles
STAT	Signal transducer and activator of transcription
TAM	Tamoxifen
TMZ	Temozolomide
TNF	Tumor necrosis factor
TPA	12-O-tetradecanoylphorbol-13-acetate
TRAP	Tartrate-resistant acid phosphatase
VCAM	Vascular cell adhesion molecules
VEGF	Vascular endothelial growth factor
WST-1	Water soluble tetrazolium salts-1
z-DEVD-fmk	Z-D (OMe)E (OMe)VD(OMe) fluoromethylketone
